# Top-Down Proteomics of Human Saliva Highlights Anti-inflammatory, Antioxidant, and Antimicrobial Defense Responses in Alzheimer Disease

**DOI:** 10.3389/fnins.2021.668852

**Published:** 2021-05-26

**Authors:** Cristina Contini, Alessandra Olianas, Simone Serrao, Carla Deriu, Federica Iavarone, Mozhgan Boroumand, Alessandra Bizzarro, Alessandra Lauria, Gavino Faa, Massimo Castagnola, Irene Messana, Barbara Manconi, Carlo Masullo, Tiziana Cabras

**Affiliations:** ^1^Dipartimento di Scienze della Vita e dell’Ambiente, Università di Cagliari, Cagliari, Italy; ^2^Dipartimento di Scienze Biotecnologiche di Base, Cliniche Intensivologiche e Perioperatorie, Università Cattolica del Sacro Cuore, Rome, Italy; ^3^Fondazione Policlinico Universitario “A. Gemelli” – IRCCS, Rome, Italy; ^4^Laboratorio di Proteomica, Centro Europeo di Ricerca sul Cervello, IRCCS Fondazione Santa Lucia, Rome, Italy; ^5^UOC Continuità Assistenziale, Fondazione Policlinico Universitario “A. Gemelli” – IRCCS, Rome, Italy; ^6^Dipartimento di Scienze Mediche e Sanità Pubblica, University of Cagliari, Cagliari, Italy; ^7^Istituto di Scienze e Tecnologie Chimiche “Giulio Natta”, Consiglio Nazionale delle Ricerche, Rome, Italy; ^8^Dipartimento di Neuroscienze, Sez. Neurologia, Università Cattolica del Sacro Cuore, Rome, Italy

**Keywords:** Alzheimer disease, salivary proteomics, S100A, cystatins, α-defensins, thymosin β4, antimicrobial peptides, oxidative stress

## Abstract

Alzheimer disease (AD) is the most prevalent neurodegenerative disease in the elderly, characterized by accumulation in the brain of misfolded proteins, inflammation, and oxidative damage leading to neuronal cell death. By considering the viewpoint that AD onset and worsening may be influenced by environmental factors causing infection, oxidative stress, and inflammatory reaction, we investigated the changes of the salivary proteome in a population of patients with respect to that in healthy controls (HCs). Indeed, the possible use of saliva as a diagnostic tool has been explored in several oral and systemic diseases. Moreover, the oral cavity continuously established adaptative and protective processes toward exogenous stimuli. In the present study, qualitative/quantitative variations of 56 salivary proteoforms, including post-translationally modified derivatives, have been analyzed by RP-HPLC-ESI-IT-MS and MS/MS analyses, and immunological methods were applied to validate MS results. The salivary protein profile of AD patients was characterized by significantly higher levels of some multifaceted proteins and peptides that were either specific to the oral cavity or also expressed in other body districts: (i) peptides involved in the homeostasis of the oral cavity; (ii) proteins acting as ROS/RNS scavengers and with a neuroprotective role, such as S100A8, S100A9, and their glutathionylated and nitrosylated proteoforms; cystatin B and glutathionylated and dimeric derivatives; (iii) proteins with antimicrobial activity, such as α-defensins, cystatins A and B, histatin 1, statherin, and thymosin β4, this last with a neuroprotective role at the level of microglia. These results suggested that, in response to injured conditions, Alzheimer patients established defensive mechanisms detectable at the oral level. Data are available via ProteomeXchange with identifier PXD021538.

## Introduction

Alzheimer disease (AD) is a neurodegenerative disease, and it is the leading cause of progressive dementia in the elderly population (McKhann et al., 2011). Neuropathologically, AD is characterized by the accumulation of amyloid beta (Aβ) plaques and by the development in the brain of neurofibrillary tangles (NFTs) composed of tau protein (Duyckaerts et al., 2009). Only a small portion of AD cases has genetic causes while the majority (95%) are sporadic and occur in people over 65 years (Scheltens et al., 2016). The sporadic form of AD is more complex since it likely results from a combination of genetic and environmental factors. Although the etiology of the disease has not been fully elucidated yet, the role of environmental factors, as a possible element of risk, has extended its relevance in AD pathology. Indeed, it has been recently hypothesized that prolonged exposure to several heavy metals (aluminium, arsenic, cadmium, lead, mercury), particulate air, some pesticides, metal-containing nanoparticles and the alteration of oral and gut microbiota may increase the risk of AD (Rahman et al., 2020). The most common consequence of exposure to environmental risk factors is the overproduction of reactive oxygen species (ROS), and several studies are converging on the fact that oxidative stress might be crucial in AD pathogenesis. Oxidative stress, observed in AD, is mainly due to Aβ misfolding (Varadarajan et al., 2000), but altered metal homeostasis, which has been demonstrated in the brain and plasma/serum of AD patients (Hozumi et al., 2011), may contribute to oxidative stress too. Nevertheless, the oral and gut microbiota-induced neuronal inflammation is a gradually emerging idea promoted by the discovery that brain infections, involving bacteria or viruses as external risk factors, can trigger Aβ deposition and AD development (Mawanda and Wallace, 2013; Hill et al., 2014).

Alzheimer disease diagnosis is heavily based on detection of Aβ and tau proteins in the central nervous system (CNS), either imaged using positron emission tomography or measured in the cerebrospinal fluid (CSF); moreover, magnetic resonance imaging can be used to measure function and reveal brain atrophy (Aisen et al., 2017). Some selective biomarkers in the CSF were shown to have excellent diagnostic accuracy (Olsson et al., 2016) such as Aβ42, which is related to the extracellular senile plaques (Ashton et al., 2018), total tau protein (T-tau), which reflects the level of neuronal damage (Scholl et al., 2019), and phosphorylated tau protein on threonine 181 (P-tau181), which correlates with tangle pathology (Hampel et al., 2010). The invasive nature of CSF collection, which limits its widespread use in routine primary care practice (Zetterberg, 2019), and the growing complexity of AD pathogenesis, highlighted the need for additional biomarkers for early preclinical diagnosis. Blood biomarkers are more easily detectable than those from the CSF for population screening; however, the use of salivary biomarkers can show more advantages. Indeed, saliva has been proposed as an easily collectable source of potential biomarkers for diagnosis and risk assessment for a range of pathological conditions that occur not only orally but also systemically (Castagnola et al., 2011; Pfaffe et al., 2011). In fact, most blood biomarkers can also be found in saliva since proteins from the blood can pass into the saliva via passive diffusion, active transport, or microfiltration (Pfaffe et al., 2011; Spielmann and Wong, 2011). It was reported that proteins from CNS are also excreted into the saliva (Spielmann and Wong, 2011), and it was suggested that the alteration of the autonomic nervous system, occurring in AD, could affect the activity of the major salivary glands and, thus, saliva production and composition (Farah et al., 2018). Recently, Gleerup et al. (2019) found that Aβ42 and tau protein could be candidates for salivary biomarkers of AD, and several proteins detectable in human saliva by proteomic approaches (Castagnola et al., 2012) have been investigated for their implication in AD, such as S100A7, S100A8, S100A9, and S100A12 (Cristóvão and Gomes, 2019) as well as cystatins C (Kaur and Levy, 2012) and B (Skerget et al., 2010). In consideration of these evidences, the present study aimed to investigate, for the first time, the salivary proteome of AD patients in comparison with HCs by a top-down proteomic approach, to evidence possible qualitative/quantitative variations associated with the disease, also in light of the eventual involvement of environmental risk factors in AD pathogenesis.

## Materials and Methods

### Reagents and Instruments

All the chemicals and reagents used for high-performance liquid chromatography separation coupled to electrospray-ion trap mass spectrometry (HPLC-ESI-IT-MS) analysis were purchased from Sigma-Aldrich (St. Louis, MO, United States). HPLC/low-resolution ESI-MS analyses were performed with a Surveyor HPLC system connected to an LCQ Advantage mass spectrometer (Thermo Fisher Scientific, San Jose, CA, United States). The chromatographic column was a Vydac C8 reverse-phase column (Grace, Hesperia, CA, United States) (2.1 × 150 mm, particle diameter 5 μm). HPLC/high-resolution ESI-MS and MS/MS experiments were carried out using an UltiMate 3000 micro HPLC apparatus (Dionex, Sunnyvale, CA, United States) equipped with an FLM-3000 flow manager module and coupled to an LTQ-Orbitrap Elite or LTQ Orbitrap XL apparatus (Thermo Fisher). The column was a Zorbax 300SB-C8 (1.0 × 150 mm; 3.5 μm particle diameter). All the chemicals and reagents for immunodetection were purchased from Bio-Rad (Hercules, California, United States); the primary mouse monoclonal antibodies (Abs) for α-defensins, cystatin A, cystatin B, S100A8, S100A9, cystatin SN, and thymosin β4 (Tβ4) were purchased from Santa Cruz Biotechnology (Dallas, TX, United States). The secondary rabbit anti-mouse Ab was from Invitrogen (Waltham, MA, United States). Standard Tβ4 was provided by Bachem (Bubendorf, Switzerland).

### Study Subjects and Clinical Data

Thirty-five subjects affected by AD (23 females and 12 males; mean age and standard deviation (SD): 80 ± 6) were recruited by the Neurology Department of the “Fondazione Policlinico Universitario A. Gemelli,” Catholic University of Rome. The clinical diagnosis of AD has been carried out according to standardized criteria (McKhann et al., 2011). The control group included 35 healthy volunteers (17 females and 18 males; mean age and SD: 78 ± 5). The informed consent process agreed with the latest stipulations established by the Declaration of Helsinki. The study was approved by the formal ethical committee of the Catholic University of Rome. Included AD patients and controls were carefully selected as non-smokers. They were not affected by any major oral disease (periodontitis, caries, or dry mouth). The clinical diagnosis of AD has been carried out according to standardized criteria (McKhann et al., 2011). Thirteen patients were classified in the moderate stage, and the remaining patients were in the mild stage. Demographic and clinical features of the included patients are reported in Supplementary Table 1 (Supplementary Material), as well as the pharmacological therapy. Nineteen patients underwent acetylcholinesterase (AChE) inhibitor therapy (donepezil 5 mg or rivastigmine 9.5 mg daily) (group G1). Eight patients were treated with AChE inhibitors in association with memantine, an antagonist of the *N*-methyl-D-aspartate receptor (NMDAR) (group G2). Eight patients were treated with memantine 20 mg daily (group G3).

### Sample Collection and Treatment

All the samples of non-stimulated whole saliva were collected between 9:00 and 12:00 a.m. Donors, in fasting conditions, were invited to sit, assuming a relaxed position, and to swallow.

Whole saliva was collected as it flowed into the anterior floor of the mouth with a soft plastic aspirator for less than 1 min and transferred to a plastic tube cooled on ice. Salivary samples were immediately diluted in a 1:1 v/v ratio with a 0.2% solution of 2,2,2-trifluoroacetic acid (TFA) containing 50 μM of leu-enkephalin as internal standard. Then, each sample was centrifuged at 20,000 *g* for 15 min at 4°C. Finally, the supernatant was separated from the precipitate and immediately analyzed by RP-HPLC-ESI-MS or stored at −80°C until the analysis for up to 2 weeks. Aliquots of 50 μl of each sample were used for the determination of the total protein concentration (TPC) by a bicinchoninic acid (BCA) assay kit (Sigma-Aldrich/Merck, Darmstadt, Germany) in duplicate, following the provided instructions.

### RP-HPLC/Low-Resolution ESI-MS Analysis

Thirty microliters of acidic extracts were injected in HPLC/low-resolution ESI-MS, applying procedures and conditions optimized in our previous studies (Contini et al., 2020; Serrao et al., 2020). The total ion current (TIC) chromatographic profiles were analyzed to selectively search and quantify the peptides/proteins reported in Supplementary Table 2 (Supplementary Material), which shows UniProtKB codes, elution times, and experimental and theoretical average mass values (Mav) of the proteins/peptides included in the study. Supplementary Table 2 also reports the multiply charged ions used for the eXtracted Ion Current (XIC) search, which were selected by excluding values common to other closely eluting proteins. A window of ± 0.5 Da was used to extract ion current peaks. Experimental Mav were obtained by deconvolution of averaged ESI-MS spectra automatically performed by using MagTran 1.0 software (Zhang and Marshall, 1998). Mav and elution times of proteins/peptides were compared with those determined on salivary samples, under the same experimental conditions, in our previous studies (Castagnola et al., 2012; Manconi et al., 2017). Experimental Mav were also compared with the theoretical ones available at the UniProtKB human data bank^1^.

### Quantification and Statistical Analysis

Quantification of peptides/proteins was performed by using the XIC peak areas measured by HPLC/low-resolution ESI-MS analysis, with the following peak parameters: baseline window 15, area noise factor 50, peak noise factor 50, peak height 15%, and tailing factor 1.5. The estimated percentage error of the XIC analysis was <8%. Eventual dilution errors occurring during sample collection were corrected by normalizing XIC peak areas of peptides/proteins with the XIC peak area of leu-enkephalin used as internal standard, as described in a previous study (Contini et al., 2020). Then, the corrected XIC peak area values of each peptide/protein were normalized on the TPC of each sample. GraphPad Prism software (version 5.0) was used to calculate means and SDs of the normalized protein XIC peak areas and to perform statistical analysis. Data distributions were tested for normality by the D’Agostino-Pearson test. A comparison between the groups of patients and controls has been performed with Mann–Whitney and Welch-corrected *t*-tests, depending on the distribution (skewed or normal) and the variance (unequal or homogeneous). Statistical analysis has been considered significant when the *p*-value was <0.05. A nonparametric ANOVA with the Kruskal–Wallis test and Dunn’s post-test was applied to compare the groups of patients treated with different pharmacological therapies.

### Preparative RP-HPLC for Salivary Cystatin B

To obtain a cystatin B-enriched sample by a preparative RP-HPLC separation, a total of 6 ml of acid-soluble fraction of human saliva from a healthy donor was injected in a HPLC system UHPL UltiMate 3,000 (Dionex, Sunnyvale, CA, United States). The chromatographic separation was carried out on an RP Vydac C8 column (Grace, Hesperia, CA, United States), 250 × 10 mm, 5 μm diameter particles, with the following eluents: A, 0.056% aqueous TFA; and B, 0.05% TFA in acetonitrile (ACN)/water 80:20 (v/v). The applied gradient was from 0 to 60% B in 40 min, from 60 to 100% in 5 min with a flow rate of 2.8 ml/min. RP-HPLC fractions collected during the separation were analyzed by HPLC-ESI-MS, and that containing the cystatin B proteoforms were lyophilized, the powder was suspended in 250 μl of 0.1% TFA, and an aliquot was subjected to a BCA assay (Sigma-Aldrich/Merck, Darmstadt, Germany) to determine the TPC to be used as standard for the immunodetection of cystatin B in the salivary pool from AD and HC groups, as explained in the following paragraph.

### Immunoblotting Analysis

For immunoblotting analysis, 25 μg of proteins from 28 AD patients (16 females and 12 males, mean age and SD: 79.7 ± 6.3) and from 28 HC (15 females and 13 males, mean age and SD: 78.4 ± 4.4) was separately mixed in pools to reach a final TPC of 10 μg/μl for both pools. Dot-blotting was applied for the immunodetection of α-defensins, cystatins A and B, S100A8, S100A9, and Tβ4. Two microliters of each pool diluted 1:10 was blotted in triplicate in a nitrocellulose membrane to detect α-defensins, S100A8, and S100A9. For detection of cystatins A and B and Tβ4, 2 μl of the 10 μg/μl concentrated pools was used. After 1 h, the membranes were incubated under stirring for 45 min in the blocking solution composed by 5% blotting-grade blocker (Bio-Rad) in TBS-T (Tris 0.02 M, NaCl 0.15 M 0.05% Tween-20, pH 7.6) and 1 h with the primary Ab (1:1,000 in TBS-T for α-defensins, S100A8, S100A9, and cystatins A and B and 1:200 for Tβ4). After washing with TBS-T, the membranes were incubated for 1 h with the secondary rabbit anti-mouse Ab horseradish peroxidase (HRP)-conjugated [IgG (H + L), 1:5,000 in TBS-T for α-defensins, S100A8, S100A9, and cystatins A and B, and IgG2b, 1:50,000 for Tβ4]. Hence, the membranes were treated with the detection solution Clarity Western ECL Substrate (Bio-Rad) according to the manufacturer’s instructions. The signals were acquired in high-sensitivity mode with the ChemiDoc MP Imaging System (Bio-Rad) and the images analyzed with the Image Lab software (4.0.1 version). The intensities of the signals, appropriately corrected by the software background subtraction, were normalized with respect to that of cystatin SN, leading to quantitatively unvaried results between patients and controls (Supplementary Table 3 and Supplementary Material). To obtain a dot-blotting in the presence of monoclonal Abs against cystatin SN, the same experimental conditions reported above were applied, except for the secondary HRP-conjugated Ab, which was a rabbit anti-mouse IgG2b, diluted 1:50,000 in TBS-T. Tβ4 signal normalization was performed with respect to the signal of 0.26 nmol of the standard peptide. Cystatin B signal normalization was performed with respect to the signal of 0.2 μg of the cystatin B-enriched sample. The full scan of the entire membranes and the results of the normalization are reported as Supplementary Material (Supplementary Figures 1–5).

### RP-HPLC/High-Resolution ESI-MS/MS Analysis

Twenty-two salivary samples (4 and 18 from the AD and HC groups, respectively) were subjected to HPLC/high-resolution ESI-MS/MS (LTQ-Orbitrap Elite or LTQ-Orbitrap XL) to perform a top-down characterization confirming the identity of the peptides and proteins investigated in this study and reported in Supplementary Table 2. The instrument operated in “Intact Protein Mode,” with the delta HCD (higher-energy collisional dissociation) vacuum pressure reduced to 0.1. The chromatographic separation was carried out using eluent A [0.1% (v/v) aqueous formic acid (FA)] and eluent B [0.1% (v/v) FA in ACN/water 80/20]. The gradient was 0–2 min 5% B, 2–10 min from 5 to 25% B (linear), 10–25 min from 25 to 34% B, 25–45 min from 34 to 70% B, and 45–55 min from 70 to 90% B at a flow rate of 50 μl/min. The injection volume was 19 μl. Full MS experiments were performed in positive ion mode with mass ranging from 400 to 2,000 *m*/*z* at a resolution of 120,000 (at 400 *m*/*z*). Capillary temperature was 275°C, source voltage 4.0 kV, and S-lens radiofrequency level 69%. In the data-independent acquisition mode, the five most abundant ions were acquired and fragmented by using collision-induced dissociation (CID) and HCD with 35% normalized collision energy for 10 ms, isolation width of 5 *m*/*z*, and activation *q* of 0.25. HPLC-ESI-MS and MS/MS data were generated by Xcalibur 2.2 SP 1.48 (Thermo Fisher Scientific, CA, United States) using default parameters of the Xtract program for the deconvolution. MS/MS data were analyzed by both manual inspection of the MS/MS spectra recorded along the chromatogram and the Proteome Discoverer 2.2 software elaboration based on the SEQUEST HT cluster as a search engine (University of Washington, licensed to Thermo Electron Corporation, San Jose, CA, United States) against the UniProtKB human data bank (188,453 entries, release 2019_03). For peptide matching, high-confidence filter settings were applied: the peptide score threshold was 2.3, and the limits were Xcorr scores >1.2 for singly charged ions and 1.9 and 2.3 for doubly and triply charged ions, respectively. The false discovery rate (FDR) was set to 0.01 (strict) and 0.05 (relaxed), and the precursor and fragment mass tolerance were 10 ppm and 0.5 Da, respectively. N-terminal pyroglutamination of Glu or Gln residues, phosphorylation on Ser and Thr residues, N-terminal acetylation, oxidation of Met and Trp residues, glutathionylation, nitrosylation, and sulfonic acid of Cys residues were selected as dynamic modifications. Because of the difficulties of the automated software in detecting with high confidence every protein and their fragments, the structural information derives in part from manual inspections of the MS/MS spectra, obtained by both CID and HCD fragmentations, against the theoretical ones generated by MS-Product software available at the Protein Prospector website^2^. All the MS/MS spectra were manually verified by using all the fragmentation spectra with an acceptable number of fragment ions. The mass spectrometry proteomics data have been deposited into the ProteomeXchange Consortium^3^ via the PRIDE (Perez-Riverol et al., 2019) partner repository with the dataset identifier PXD021538.

## Results

The results obtained in the present study concern the fraction of salivary peptides and proteins soluble in acidic solution and directly analyzable by RP-HPLC-ESI-MS by a top-down approach. The investigated components belong to the following protein families: acidic proline-rich proteins (aPRPs); statherin; histatins (Hst); salivary cystatins (S-type); cystatins A, B, C, and D; α-defensins; Tβ4; antileukoproteinase (SLPI); S100A proteins; and all the variants and the posttranslational modifications (PTMs) that we have characterized in human saliva by our approach (Supplementary Table 2). Overall, 56 proteoforms were investigated in each salivary sample, including modified proteoforms generated by phosphorylation, proteolysis, N-terminal acetylation, methionine or tryptophan oxidation, and cysteine oxidation (formation of disulfide bridges, glutathionylation, cysteinylation, and nitrosylation) (Supplementary Table 2). Protein/peptide levels, measured by MS analysis with a standardized XIC procedure and normalized with respect the TPC, were compared between AD patients and controls. TPC was lower in AD patients (807 μg/ml ± 544) than in controls (1,054 μg/ml ± 445) with a *p*-value of 0.01. Proteins/peptides showing significantly different levels between the AD and HC groups are reported in Table 1, while the results of the statistical analysis of proteins/peptides with similar abundance in the two groups are reported as Supplementary Material (Supplementary Table 3).

**TABLE 1:** XIC peak areas values (mean ± SD) normalized on total protein concentration and frequencies of salivary proteins/peptides resulted to levels significantly different between the AD group and HC group.

Protein/peptide	XIC peak areas × 10^5^ (mean ± SD) and frequency		*p*-Values
	AD	HC	AD vs HC
P-C peptide	9.8 ± 7.7 (33/35)	5.9 ± 4.4 (34/34)	*p* = 0.02 AD↑
Statherin 2P	9.7 ± 9.6 (35/35)	1.1 ± 0.9 (33/34)	*p* < 0.0001 AD↑
Stath. desF_43_	1.5 ± 1.3 (35/35)	0.4 ± 0.7 (33/34)	*p* < 0.0001 AD↑
Stath. des1–9	0.6 ± 0.6 (29/37)	0.08 ± 0.1 (24/34)	*p* < 0.0001 AD↑
Stath. des1–13	0.2 ± 0.3 (24/35)	0.08 ± 0.1 (16/34)	*p* = 0.01 AD↑
Hst-1 1P	1.6 ± 1.5 (33/35)	0.8 ± 0.8 (28/34)	*p* = 0.01 AD↑
Hst-1 0P	0.3 ± 0.4 (24/35)	0.1 ± 0.2 (17/34)	*p* = 0.02 AD↑
Cyst A	1.9 ± 1.6 (32/35)	1.0 ± 0.9 (29/34)	*p* = 0.007 AD↑
Cyst B-SSG	0.9 ± 1.2 (29/35)	0.3 ± 0.3 (27/34)	*p* = 0.006 AD↑
Cyst-B-SSC	0.2 ± 0.3 (20/35)	0.08 ± 0.1 (18/34)	•
Cyst B-SS dimer	0.6 ± 0.5 (31/35)	0.2 ± 0.3 (27/34)	*p* = 0.0006 AD↑
Cyst B tot	1.7 ± 1.9 (33/35)	0.6 ± 0.6 (27/34)	*p* = 0.002 AD↑
α-defensin 1	1.8 ± 2.0 (32/35)	0.5 ± 0.6 (24/34)	*p* = 0.0001 AD↑
α-defensin 2	1.2 ± 1.3 (30/35)	0.4 ± 0.4 (27/34)	*p* = 0.0009 AD↑
α-defensin 3	0.6 ± 0.5 (25/35)	0.2 ± 0.3 (17/34)	*p* = 0.0003 AD↑
α-defensin 4	0.2 ± 0.3 (13/35)	0.05 ± 0.1 (11/34)	*p* = 0.01 AD↑
α-defensin tot	3.9 ± 4.0 (32/35)	1.2 ± 1.5 (24/34)	*p* = 0.0005 AD↑
Tβ4	0.7 ± 0.8 (25/35)	0.2 ± 0.4 (16/34)	*p* = 0.01 AD↑
S100A8	0.6 ± 1.3 (9/35)	0.04 ± 0.2 (2/34)	*p* = 0.02 AD↑
S100A8 hyperoxidized	0.2 ± 0.4 (5/35)	0.04 ± 0.2 (3/34)	•
S100A8-SNO	0.8 ± 1.8 (12/35)	Not detected	NA
S100A8 tot	1.6 ± 2.7 (22/35)	0.08 ± 0.2(5/34)	*p* < 0.0001 AD↑
S100A9(S) (all proteoforms)	3.3 ± 3.2 (32/35)	0.5 ± 0.8 (16/34)	*p* < 0.0001 AD↑
S100A9(L)-SSG	1.5 ± 2.4 (22/35)	0.3 ± 0.4 (16/34)	*p* = 0.004 AD↑
S100A9 tot	4.8 ± 5.4 (33/35)	0.9 ± 1.2 (19/35)	*p* < 0.0001 AD↑

*(•), non-statistically significant *p*-values. (NA), absence of a protein.*

Higher levels in AD group than in HC were observed for statherin and three of its naturally occurring fragments (desF43, des1–9, and des1–13), for the P-C peptide, and for Hst-1 proteoforms, both phosphorylated and non-phosphorylated (Table 1). No changes in the abundance of other secretory proteins/peptides reported in Supplementary Table 2 were observed.

Alzheimer disease patients showed also significantly higher levels of α-defensins 1–4, Tβ4, cystatin A, and the dimeric and glutathionylated proteoforms of cystatin B (Table 1). Moreover, the total S100A8 was more abundant in AD than in HC. The unmodified proteoform of S100A8 was observed in only 9 AD patients and in 2 HC subjects. In five AD patients, S100A8 was observed just as a hyperoxidized form (carrying Met_1/78_ and Trp_54_ oxidation and Cys_42_ oxidized to sulfonic acid). The nitrosylated proteoform at Cys_42_ (S100A8-SNO) was observed only in 12 AD patients, and only in 7 of these patients was the unique proteoform detected. All the proteoforms of S100A9(S) were more abundant in AD patients, and thus Table 1 reports the sum of the XIC peak areas of all the S100A9(S) proteoforms (short and short phosphorylated at Thr_108_ and their correspondent oxidized derivatives at Met_89_ or Met_78_ or Met_76_ or Met_58_, Supplementary Table 2). Finally, the long glutathionylated S100A9 was significantly more abundant in the AD group than in the HC group.

**FIGURE 1:**
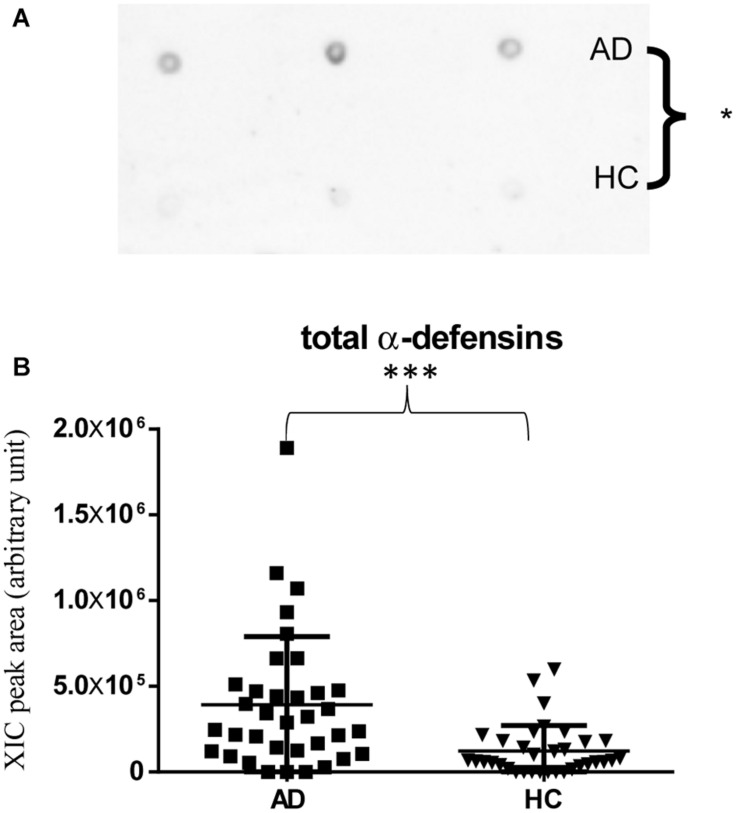
Dot-blotting immunodetection of total α-defensins in salivary pools of AD and HC samples **(A)** and distribution plot of the XIC peak area values measured by HPLC-ESI-MS of total α-defensins **(B)** in each salivary sample from the AD and HC groups (XIC peak areas of the four identified defensins were considered in the sum, see Supplementary Table 2) (^∗^, *p* < 0.05; ^∗∗∗^, *p* < 0.001).

**FIGURE 2:**
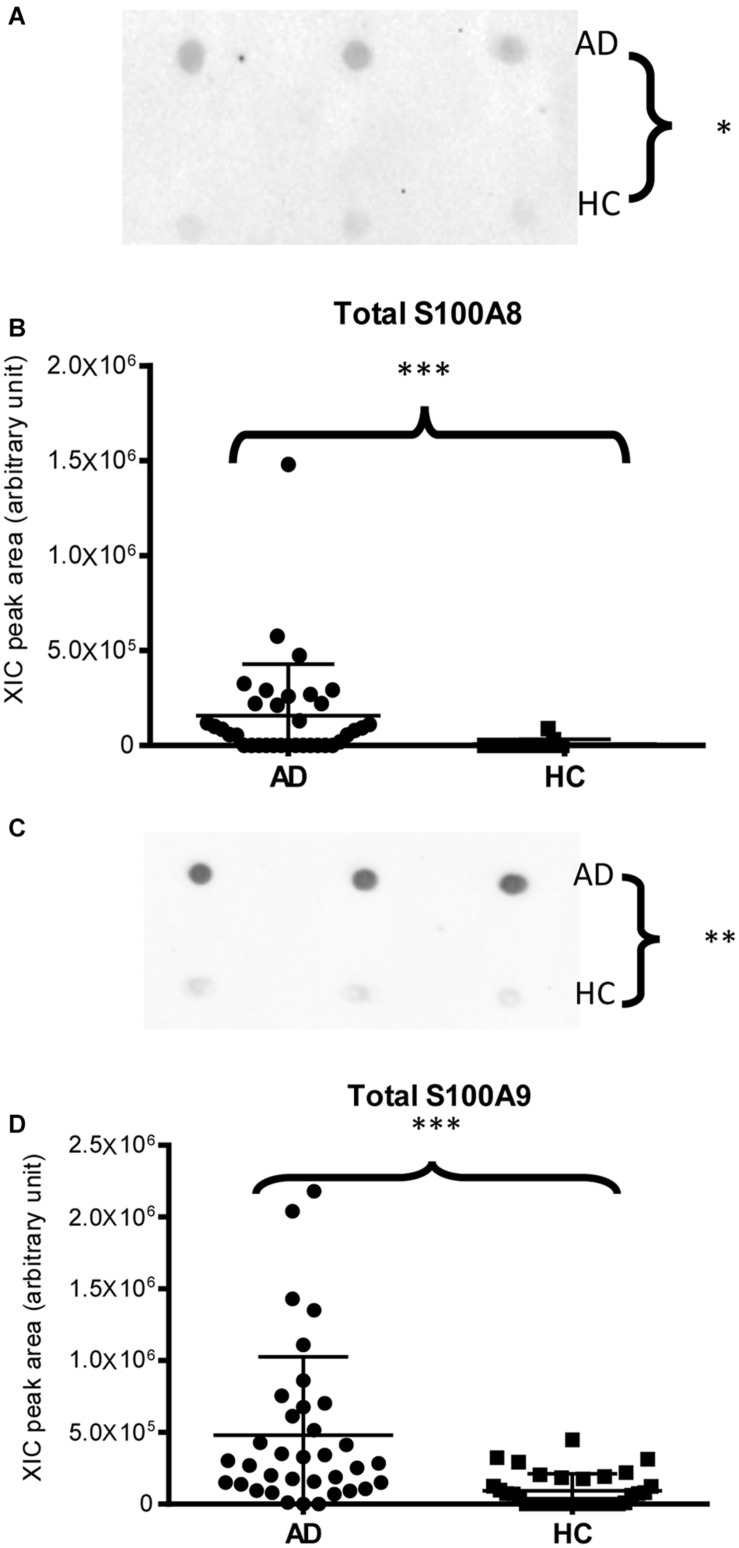
Dot-blotting immunodetection of total S100A8 **(A)** and total S100A9 **(C)** in salivary pools of AD and HC samples and distribution plot of the XIC peak area values of total S100A8 **(B)** and total S100A9 **(D)** measured by HPLC-ESI-MS in each salivary sample from the AD and HC groups (XIC peak areas of all the identified proteoforms of S100A8, such as S100A9, were considered in the sum, see Supplementary Table 2) (^∗^, *p* < 0.05; ^∗∗^, *p* < 0.01; ^∗∗∗^, *p* < 0.001).

**FIGURE 3:**
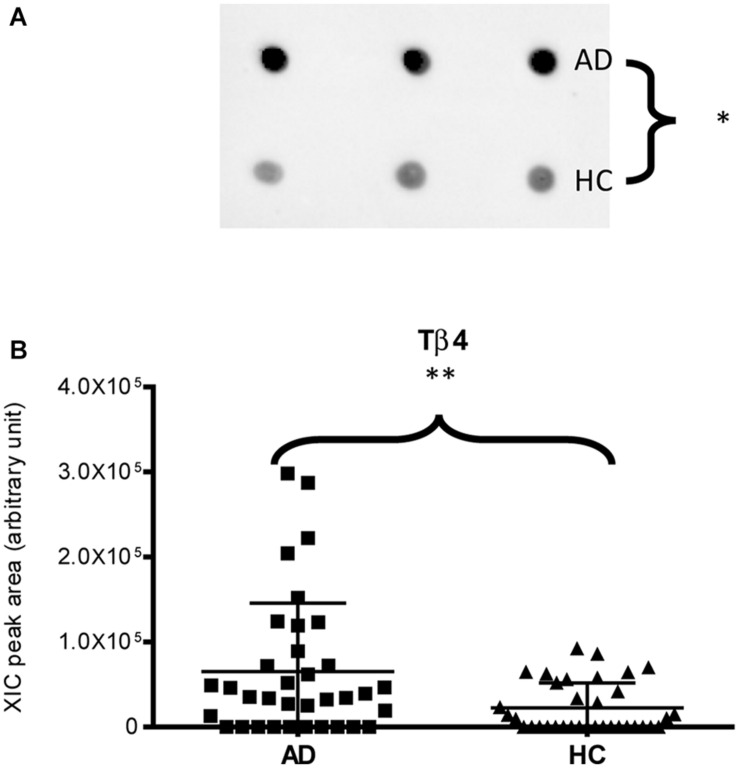
Dot-blotting immunodetection of Tβ4 in salivary pools of AD and HC samples **(A)** and distribution plot of the XIC peak area values of Tβ4 measured by HPLC-ESI-MS **(B)** in each salivary sample from the AD and HC groups (^∗^, *p* < 0.05; ^∗∗^, *p* < 0.01).

Immunodetection approaches confirmed some of the MS results. Dot-blotting analysis, shown in Figure 1A and Supplementary Figures 1A,B, evidenced a higher abundance of total α-defensins in AD samples than in HC salivary samples (*p* = 0.02), in accordance with the MS data obtained considering the sum of the XIC peak area values of all the α-defensins in each sample (*p* = 0.0005) (Figure 1B). Similar results were obtained for S100A8 and S100A9, and also in this case, dot-blotting was used to immunodetect total S100A8 (Figure 2A) and total S100A9 (Figure 2C). The signal intensity of total S100A8 was significantly higher in the AD salivary pool (*p* = 0.03) than in the HC one (Supplementary Figure 2A), in accordance with MS data obtained on total S100A8 (Figure 2B). Analogously, total S100A9 (short and long proteoforms) exhibited a signal intensity higher in the AD salivary pool than in the HC one (*p* = 0.007) (Figure 2C and Supplementary Figure 3). The result of the dot-blotting was in accordance with the MS results obtained on the total S100A9 (*p* < 0.0001) (Figure 2D). Similarly, the immunodetection of Tβ4 confirmed the highest levels of the peptide in AD patients (*p* = 0.02) obtained by the MS approach (Figures 3A,B).

**FIGURE 4:**
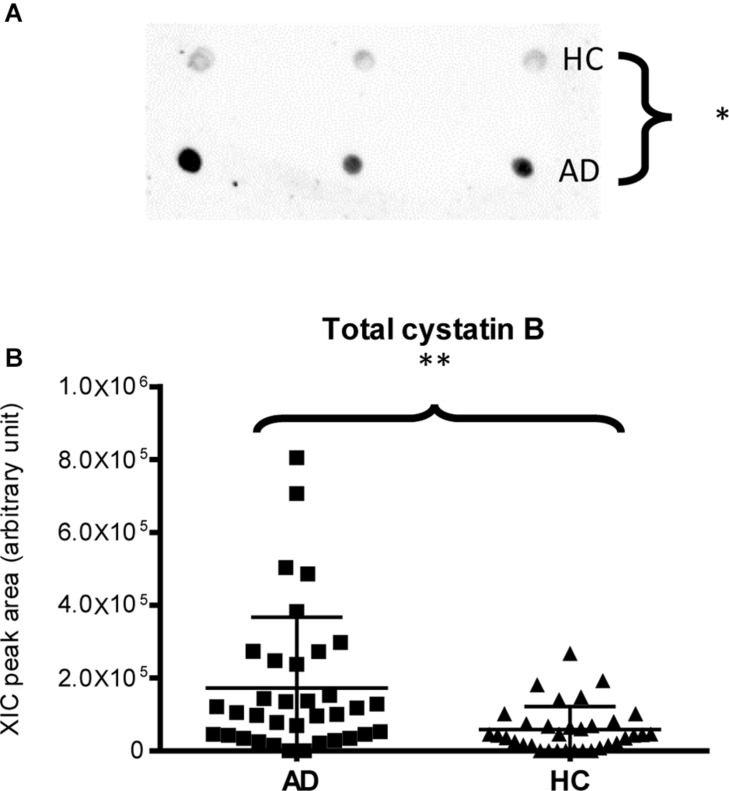
Dot-blotting immunodetection of total cystatin B in salivary pools of AD and HC samples **(A)** and distribution plot of the XIC peak area values of total cystatin B measured by HPLC-ESI-MS **(B)** in each salivary sample from the AD and HC groups (XIC peak areas of the three identified proteoforms of cystatin B were considered in the sum, see Supplementary Table 2) (^∗^, *p* < 0.05; ^∗∗^, *p* < 0.01).

Finally, dot-blotting experiments showed more abundance of total cystatin B (dimeric and monomeric proteoforms) in AD patients than in HC (*p*-value = 0.03, Figure 4A), confirming the MS results (Figure 4B). Immunodetection of cystatin A did not provide reliable results (data not reported), probably because the cystatin A levels were under the sensitivity limits of our method.

The comparison among the three AD patient groups treated with different pharmacological therapies, named G1, G2, and G3, was limited by the very low number of subjects of the G2 and G3 groups and thus provided results that we consider preliminary and report in the Supplementary Materials (Supplementary Figure 6 and Supplementary Table 4). These data highlighted similar TPC values in the three patient groups. The ANOVA showed that patients in the G1 and G2 groups, rather than G3, could contribute mainly to the highest levels of the peptides and proteins reported in Table 1, with the exception of statherin and its proteoforms found abundant also in the G3 group and P-C peptide, for which G3 was the group with the highest values.

## Discussion

Saliva, as a mirror of oral and systemic health, provides valuable information because it contains not only proteins specifically secreted by the salivary glands (Messana et al., 2008) but also proteins derived from the gingival crevicular fluid (Ghallab, 2018; Iavarone et al., 2020) from oral microflora (Grassl et al., 2016) and plasmatic proteins transported from blood to saliva by both intracellular and extracellular pathways. The present work evidenced increased levels of some proteins and peptides both secreted and not secreted by the salivary glands from AD patients compared to HCs. Among the proteins not secreted by the salivary glands, this study highlighted a higher level of proteins with a multifaceted nature, namely, S100A8 and S100A9, α-defensins 1–4, Tβ4, and cystatins A and B. They are involved in many biological functions also at the level of the nervous system, and thus, it is not surprising that they may be implicated in molecular processes associated with AD pathogenesis.

S100A8 and S100A9 mainly derive from neutrophils and macrophages, which participate in inflammatory process. During inflammation, S100A8 and S100A9 are actively released and exert a critical role in modulating the inflammatory response by stimulating leukocyte recruitment and inducing cytokine secretion. S100A8 and S100A9 may play a dual role in inflammation, and their pro-inflammatory activity can switch to anti-inflammatory probably depending on the local microenvironment (El Gazzar, 2015). Our results did not allow us to establish if the increased levels of S100A9 and S100A8 observed in saliva of AD patients were linked to a pro- or anti-inflammatory role; however, they agree with studies performed by other research groups. Indeed, as a consequence to neuroinflammations, overexpression of S100A proteins had already been observed in AD where they seem to be involved in several processes related to APP processing, Aβ levels, tau protein PTMs, formation of protein inclusions, and multiple signaling pathways (Cristóvão and Gomes, 2019). S100A8 was found to be upregulated in the sera of AD patients (Shen et al., 2017) and in the hippocampus of mice models of AD (Lodeiro et al., 2017). Similarly, S100A9 was found to be strongly increased in brain lysates of both AD patients and AD mice (Chang et al., 2012; Kummer et al., 2012) and also in activated microglia and neurons with tau NFTs (Shepherd et al., 2006). Several studies evidenced a strong positive correlation between S100A9 levels and AD pathology: knockdown of S100A9 improved cognition on model mice of AD and reduced global levels of Aβ and APP C-terminal fragments due to decreased activity of the beta-site amyloid precursor protein cleaving enzyme 1 (BACE-1) (Chang et al., 2012). Moreover, a correlation between S100A9 and calcium dysregulation in AD has been also evidenced since knockdown of S100A9 significantly diminished the increase of Ca^2+^ levels determined by APP C-terminal fragments or by Aβ (Ha et al., 2010).

Our study on saliva from AD patients highlighted, with respect to HC, higher levels not only of S100A8 and S100A9 but also of their oxidized proteoforms, namely, the hyperoxidized proteoform of S100A8, S100A8-SNO, and glutathionylated long S100A9. Oxidative modifications of proteins are common in neurological disorders since the extracellular milieu is strongly oxidizing as a result of generation of ROS and reactive nitric oxide species (RNS) (Smith et al., 1997; Duncan and Heales, 2005). Various environmental stimuli are responsible for ROS/RNS production by either inducing neuroinflammation or stimulating/preventing several signaling pathways, which leads to increased or reduced enzyme activity that boosts the addition of oxidative products in neuronal cells (Rahman et al., 2020). Indeed, high concentrations of Fe^3+^ in NFTs and Aβ aggregates increased the levels of H_2_O_2_ and advanced glycation end products (Smith et al., 1997). Furthermore, it is known that Aβ chemotaxis of microglia and amyloid fibril phagocytosis represent inflammatory stimuli and play a critical role in ROS production (Wang et al., 2015). Also, the NADPH oxidase system has been identified as an important source of intracellular ROS, thus playing a key role in the generation of oxidative stress in neurodegenerative diseases (Tarafdar and Pula, 2018). Therefore, it is worth underlining that S100A8 and S100A9 themselves, increasing intracellular NADPH oxidase activity, give a contribution to ROS generation (Benedyk et al., 2007). Oxidation may represent a switch, whereby the modified proteins display anti-inflammatory functions, such as that demonstrated for S100A8-SNO (Lim et al., 2008); S100A9 and S100A8 are able also to act as ROS/RNS scavengers; in particular, S100A8 is sensitive to oxidative cross-linking and massive oxidation and shows capacity to reduce oxidative damage (Goyette and Geczy, 2011). Changes in the inflammatory microenvironment, PTMs of S100A8 and S100A9, binding with transition metals (Zn^2+^ and Ca^2+^), and S100A8/S100A9 heterodimer formation most likely play an important role in the functional switching of these pleiotropic proteins (El Gazzar, 2015).

In addition to exposure to oxidative stress, the microbiota-induced neuronal inflammation is a gradually emerging concept promoted by the discovery that brain infections, involving external risk factors such as bacteria or viruses, can trigger Aβ deposition and AD development (Mawanda and Wallace, 2013). In fact, chronic brain infections, caused by various pathogens, have been reported for AD patients (Alonso et al., 2014; Lövheim et al., 2015). In particular, it has been hypothesized that oral and gut microbiota, or their released endotoxins, may alter the permeability of the blood–brain barrier (BBB), facilitate the cerebral colonization by opportunistic pathogens, and induce microglia activation, resulting in increased levels of pro-inflammatory cytokines, which lead to neuronal loss and neurodegeneration (Wang et al., 2015). The inflammatory response thereafter may indirectly lead to the upregulation of Aβ production (Welling et al., 2015). It has been supposed that neuropathological alterations might be associated with abnormal expression and/or regulation of antimicrobial peptides, including defensins (Williams et al., 2012), and it is worth noting that Aβ itself exerted antimicrobial activity against bacteria and viruses (Soscia et al., 2010). Such antimicrobial peptides open an intriguing prospect for the detection and follow-up of such cerebral infection since they, as part of the innate immune system, can efficiently penetrate the BBB and target microbes. Besides their main role as antibacterial and antiviral peptides, defensins also exert numerous immunological effects (Oppenheim and Yang, 2005). Our study evidenced higher levels of α-defensins 1–4 in the saliva of AD patients than in HCs. In accordance with our results, Watt et al. (2015) demonstrated that the levels of α-defensins 1–3 were elevated in both CSF and sera of AD patients, indicating that an inflammatory condition is present not only at brain level but also in other body districts.

It is fascinating in the contexts of AD also, the antimicrobial and anti-inflammatory activities of thymosin β4 (Carion et al., 2020) that was found to be more abundant in the saliva of AD patients than in HCs. Tβ4 is the most abundant Tβ in human tissues, including CNS, where it exerts multiple biological functions, such as downregulation of inflammatory chemokines and cytokines, promotion of cell migration, blood vessel formation, cell survival, stem cell maturation, inhibition of microbial growth, and antiapoptotic factor on gingival fibroblasts (Hannappel, 2007), as well as neuroprotective and neuroregenerative effects (Chopp and Zhang, 2015; Zhang et al., 2020). The increased level of Tβ4 in saliva from AD patients is interesting and probably reflects an increase in the brain, in view of the elevated levels of Tβ4 found in reactive microglia of AD patients, where it suppresses the pro-inflammatory signaling (Zhang et al., 2020).

Interestingly, the saliva of AD patients was also characterized by increased levels of cystatin B, which is widely expressed in numerous tissues, including the brain, and of cystatin A, expressed mainly in epidermal cells, lymphoid tissues, and oral squamous epithelia (Magister and Kos, 2013). It has been suggested that cystatins could play a role in AD; indeed, cystatins A and B have been reported to localize to amyloid plaques of various origins (Ii et al., 1993; Bernstein et al., 1994). It is interesting to underline that cystatins B and A, like cystatin C, are considered potential Aβ-binding proteins *in vitro* (Skerget et al., 2010), able to break down amyloid aggregates in cells, and for this reason, they are also called “amateur chaperones.” Indeed, it was demonstrated that cystatin B can bind Aβ and inhibit its fibrillization. Binding is dependent on the oligomeric state (Skerget et al., 2010) of cystatin B, with the tetramer showing the highest affinity. Unfortunately, we were unable to determine the oligomeric state of cystatin B in saliva. This opens a new perspective for further studies on the salivary proteome of AD patients. Cystatins A and B may also play an important role as regulation factors of inflammation through the inhibition of cathepsins (Soond et al., 2019). In particular, cystatin B, the main natural inhibitor of cathepsin B, may exert a neuronal protective key role in AD. In fact, it should be highlighted that the chronic systemic exposure to lipopolysaccharide produced by periodontal bacteria may result in an overexpression of cathepsin B, which has been recently demonstrated to play a critical role in initiating neuroinflammation and neural dysfunction (Wu et al., 2017). Cathepsin B is a beta secretase enzyme, and similar to the BACE-1, it cleaves APP at the beta-cleavage site, creating amyloid fragments (Hook et al., 2011). Besides the principal role of cystatin B as an inhibitor of cathepsin B, recent data show additional interesting roles in maintenance of cell homeostasis, reduction of oxidative stress (Butinar et al., 2014), and prevention of apoptosis (Sun et al., 2012).

Saliva of AD patients compared to the HC group also showed higher levels of some peptides secreted by the salivary glands that are involved in the metal homeostasis and defense of the oral cavity, namely, histatin-1, both phosphorylated and not phosphorylated, and statherin. Histatin-1 is a peptide with multifaceted actions, including bacterial and host protease inhibitory properties, wound healing processes, and stimulation of cell migration (Torres et al., 2018). Moreover, histatin-1, similar to cystatin B, is able to inhibit bacterial lipopolysaccharide activity and consequently inflammatory cytokines and production of other pro-inflammatory factors (Sugiyama, 1993). Indeed, histatin-1 together with other salivary Hst, defensins and cathelicidins, represent the main family of antimicrobial peptides in mammals (Wang, 2014). Histatins carry two metal-binding motifs in the N-terminal domain, by which they exert their antimicrobial action: the Cu(II)/Ni(II) binding (ATCUN) motif and the Zn(II)-binding motif HEXXH (Melino et al., 2014). Interestingly, Huang et al. (1997) evidenced the presence of a similar Zn(II)-binding motif (EXHH) in Aβ1–40 amyloid peptides. Antibacterial activities against oral pathogens have been demonstrated also for statherin and its C-terminal fragments (Kochańska et al., 2000), which, in particular, retain the specific binding sites for *Porphyromonas gingivalis*, the key-stone pathogen in periodontitis (Amano et al., 1996). The increased level of these antimicrobial peptides in saliva of AD patients can be linked to an oral dysbiosis, which is often observed in such patients. It is worth noting that recent epidemiological studies have identified a strong association between the periodontal pathogen *P. gingivalis* and neurodegeneration. *P. gingivalis* has been detected in the brain autopsy and CSF of individuals diagnosed with AD (Ryder, 2020). Another recent study demonstrated the presence of *P. gingivalis*-derived lipopolysaccharides in brain samples from AD patients (Poole et al., 2013) and the presence of gingipains, a class of *P. gingivalis* proteases, in neurons, tau tangles, and Aβ of individuals with AD (Dominy et al., 2019). Oral dysbiosis in elderly people with AD was frequently observed as well as the propensity to periodontal problems; AD individuals may also suffer from xerostomia and oral lesions, such as stomatitis and candidiasis (Mancini et al., 2010). These conditions have been associated with a decrease of submandibular salivary flow in patients with AD without medications (Ship et al., 1990), which is considered an effect of the disease itself or associated with the anticholinergic therapy often applied (Mancini et al., 2010). Nevertheless, the results obtained in the present study did not appear linked to pathological conditions occurring in the oral cavity, since the enrolled patients were selected on the basis of absence of manifest oral diseases. Moreover, our results could not be considered a consequence of glandular flow variations due to the therapy; indeed, no statistically significant differences in the TPC were measured among the three (G1, G2, and G3) groups of patients with diverse drug treatments. Additionally, among all the peptides/proteins of glandular origins, only some peptides, such as statherin, histatin 1, and P-C peptides, changed their abundance in the saliva of AD patients with respect to the controls. This evidence suggested a connection between the attendance of the AD and the overexpression and/or oversecretion of these specific peptides. Finally, preliminary results from the comparison among G1, G2, and G3 groups appeared to display that almost all proteoforms found at high levels in the AD saliva were more concentrated in G1 and G2, treated with AChE inhibitors, than in G3 group; this last group, treated with an NMDAR antagonist, showed levels similar to those of the HC group. Only statherin proteoforms and P-C peptides were found at the highest levels in the G3 group. A further investigation focused on implications of the pharmacological therapy is desirable with a greater number of patients.

The data collected in the present study did not allow us to demonstrate that infections and/or inflammation occurred in our AD patients at the moment of the sample collection or to affirm that our results were a consequence of previous injury events. Certainly, we observed that peptides and proteins involved in the innate immunoprotection, both specific of the oral cavity and also expressed in other body districts, were abundant in salivary samples of AD patients, particularly proteins acting as ROS/RNS scavengers and with a neuroprotective role, such as S100A8, S100A9, and cystatin B; proteins with antimicrobial activity, such as α-defensins, cystatins A and B, histatin-1, statherin, and Tβ4; and peptides involved in the homeostasis of the oral cavity. Almost all these peptides/proteins are able to act as anti-inflammatory factors, and among these, Tβ4 has an important regulatory role at the microglia level. Moreover, several experimental and clinical data confirm a key role of oral and gut dysbiosis in neurodegeneration. The merging of oral-/gut-derived inflammatory response together with aging and poor diet in the elderly may contribute to the pathogenesis of AD. The results obtained in this study support the view that, in the AD patients under study, several protective mechanisms against pathogen-targeting agents generating ROS and RNS, infections, and inflammatory conditions have been established.

## Data Availability Statement

The datasets presented in this study can be found in online repositories. The names of the repository/repositories and accession number(s) can be found below: https://www.ebi.ac.uk/pride/archive/, PXD021538.

## Ethics Statement

The studies involving human participants were reviewed and approved by the Ethical Committee of the Catholic University of Rome. The patients/participants provided their written informed consent to participate in this study.

## Author Contributions

CC, AO, and CD: MS data analysis. CC, SS, FI, and MB: MS/MS data analysis. CC, TC, BM, and AO: statistical analysis. CC, SS, and CD: immunodetection experiments. TC, BM, CM, MC, and IM: study design. TC, BM, IM, AO, GF, MC, and CM: manuscript writing. CM, AM, AB, and AL: clinical data collection and patient diagnosis. All authors contributed to the article and approved the submitted version.

## Conflict of Interest

The authors declare that the research was conducted in the absence of any commercial or financial relationships that could be construed as a potential conflict of interest.

## Footnotes

1
https://www.expasy.org/

2
http://prospector.ucsf.edu/prospector/mshome.htm

3
http://www.ebi.ac.uk/pride
