# Corrigendum: Top-Down Proteomics of Human Saliva Highlights Anti-inflammatory, Antioxidant, and Antimicrobial Defense Responses in Alzheimer Disease

**DOI:** 10.3389/fnins.2021.743596

**Published:** 2021-08-13

**Authors:** Cristina Contini, Alessandra Olianas, Simone Serrao, Carla Deriu, Federica Iavarone, Mozhgan Boroumand, Alessandra Bizzarro, Alessandra Lauria, Gavino Faa, Massimo Castagnola, Irene Messana, Barbara Manconi, Carlo Masullo, Tiziana Cabras

**Affiliations:** ^1^Dipartimento di Scienze della Vita e dell'Ambiente, Università di Cagliari, Cagliari, Italy; ^2^Dipartimento di Scienze Biotecnologiche di Base, Cliniche Intensivologiche e Perioperatorie, Università Cattolica del Sacro Cuore, Rome, Italy; ^3^Fondazione Policlinico Universitario “A. Gemelli” – IRCCS, Rome, Italy; ^4^Laboratorio di Proteomica, Centro Europeo di Ricerca sul Cervello, IRCCS Fondazione Santa Lucia, Rome, Italy; ^5^UOC Continuità Assistenziale, Fondazione Policlinico Universitario “A. Gemelli” – IRCCS, Rome, Italy; ^6^Dipartimento di Scienze Mediche e Sanità Pubblica, University of Cagliari, Cagliari, Italy; ^7^Istituto di Scienze e Tecnologie Chimiche “Giulio Natta”, Consiglio Nazionale delle Ricerche, Rome, Italy; ^8^Dipartimento di Neuroscienze, Sez. Neurologia, Università Cattolica del Sacro Cuore, Rome, Italy

**Keywords:** Alzheimer disease, salivary proteomics, S100A, cystatins, α-defensins, thymosin β4, antimicrobial peptides, oxidative stress

In the original article, the Supplementary Table 4 and the Supplementary Figure 6, cited on page 6 in the original article, are missing from the Supplementary Materials. The corrected Supplementary Table 4 and Supplementary Figure 6 are shown below. The Supplementary Material of the original article has been updated.

**Table S4:** XIC peak areas values (mean ± SD) normalized on TPC, frequencies and p-values obtained by statistical analysis by comparing the three patients' groups treated with different therapies by non-parametric ANOVA with the Krustal-Wallis test and Dunn's post test. p-values > 0.05 are not statistically significant (∙).

**Peptide**	**XIC Peak Areas x 10** ^****5****^ **(mean** **±** **SD) and Frequency**				
	**G1 (nr 19)**	**G2 (nr 8)**	**G3 (nr 8)**	**HC (nr 34)**	**ANOVA p value**
α-defensin 1	2.2 ± 2.3 (19)	2.3 ± 1.8 (8)	0.6 ± 0.8 (5)	0.5 ± 0.6 (24)	p <0.0001 (G1vs G3*, G2 vs G3*, G1 vs HC***, G2 vs HC**)
α-defensin 2	1.5 ± 1.5 (19)	1.5 ± 1.2 (7)	0.4 ± 0.6 (5)	0.4 ± 0.4 (27)	p = 0.0003 (G1vs G3*, G1 vs HC**, G2 vs HC*)
α-defensin 3	0.7 ± 0.5 (16)	0.8 ± 0.5 (8)	0.2 ± 0.4 (2)	0.2 ± 0.3 (17)	p <0.0001 (G1vs G3*, G2 vs G3*, G1 vs HC***, G2 vs HC**)
α-defensin 4	0.2 ± 0.3 (8)	0.4 ± 0.2 (8)	0.04 ± 0.08 (2)	0.05 ± 0.1 (11)	p = 0.0004 (G1vs G2*, G2 vs G3**, G2 vs HC***)
Tβ4	0.5 ± 0.6 (14)	1.1 ± 0.9 (8)	0.2 ± 0.4 (3)	0.2 ± 0.4 (16)	p = 0.002 (G2vs G3**, G2 vs HC**)
Stath. 2P	8.8 ± 7.7 (19)	5.9 ± 4.4 (6)	11.0 ± 7.1 (8)	1.1 ± 0.9 (33)	p <0.0001 (G1 vs HC***, G2vs HC*, G3 vs HC***)
Stath. Des1-9	0.5 ± 0.6 (16)	0.6 ± 0.9 (6)	0.8 ± 0.6 (7)	0.08 ± 0.1 (24)	p = 0.0002 (G1 vs HC**, G3 vs HC**)
P-C peptide	9.3 ± 5.1 (19)	8.6 ± 12.6 (6)	12.3 ± 6.9 (8)	5.6 ± 4.4 (34)	p = 0.01 (G3 vs HC*)
Cyst. B tot	2.0 ± 2.2 (18)	1.9 ± 1.6 (8)	0.9 ± 1.2 (7)	9.3 ± 5.1 (27)	p = 0.006 (G1vs HC*, G2 vs HC*)
Cyst A	1.8 ± 1.6 (17)	2.9 ± 1.9 (8)	1.2 ± 0.9 (7)	1.0 ± 0.9 (29)	p = 0.02 (G2 vs HC*)
S100A8 tot	1.1 ± 1.5 (12)	0.9 ± 1.1 (6)	0.4 ± 0.5 (4)	0.08 ± 0.2(5)	p <0.0001 (G1 vs HC***, G2 vs HC**)
S100A8-SNO	1.1 ± 2.3 (9)	0.7 ± 1.0 (3)	Not detected	Not detected	p <0.0001 (G1 vs G3*, G1 vs HC***)
S100A9(S) tot	1.1 ± 1.5 (18)	0.9 ± 1.1 (7)	0.4 ± 0.5 (7)	0. .5 ± 0.8 (16)	p <0.0001 (G1 vs HC***, G2 vs HC*)

**Supplementary Figure 6:**
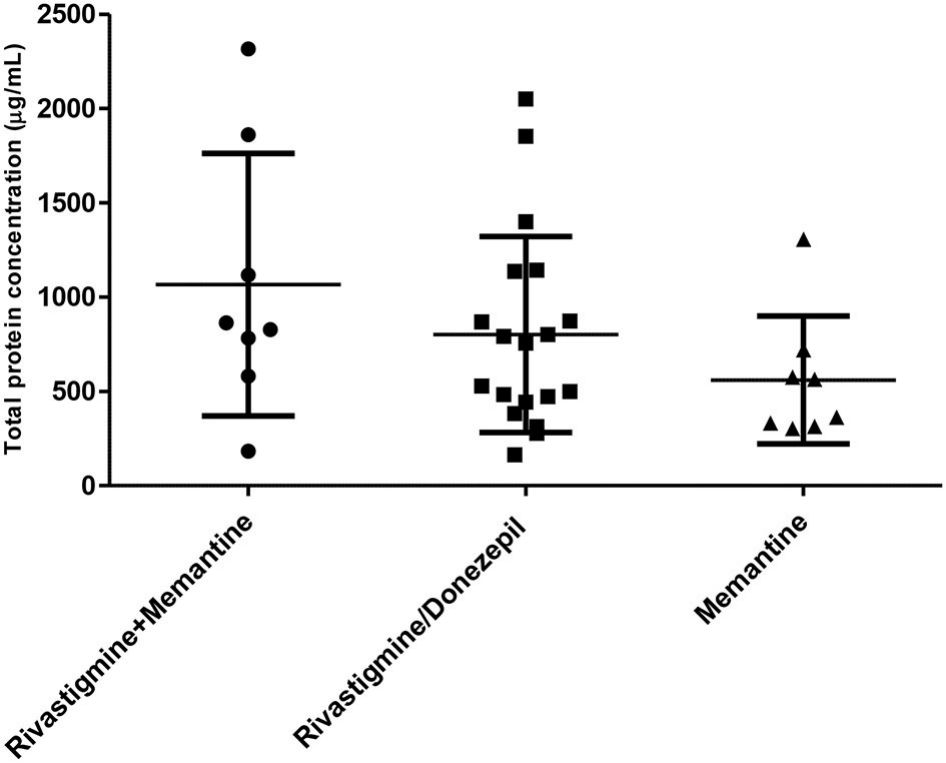
Total protein concentration measured in the subgroups of patients with different pharmacological treatment.

The authors apologize for this error and state that this does not change the scientific conclusions of the article in any way. The original article has been updated.

## Publisher's Note

All claims expressed in this article are solely those of the authors and do not necessarily represent those of their affiliated organizations, or those of the publisher, the editors and the reviewers. Any product that may be evaluated in this article, or claim that may be made by its manufacturer, is not guaranteed or endorsed by the publisher.

