# Supplementary material for: Top-Down Proteomics of Human Saliva Highlights Anti-inflammatory, Antioxidant, and Antimicrobial Defense Responses in Alzheimer Disease
Source: Front Neurosci. 2021 May 26;15:668852. doi: 10.3389/fnins.2021.668852 (PMC8189262; doi:10.3389/fnins.2021.668852)

Supplementary Material

1 Supplementary Tables

Table S1. Demographic and clinical features and pharmacological treatment of the patients included in the study.

Patients	Sex and age	Diagnosis	Pharmacological treatment
#1	M, 82	AD (moderate)	Rivastigmine + Memantine
#2	F, 80	AD (moderate)	Rivastigmine + Memantine
#3	M, 85	AD (mild)	Rivastigmine
#5	F, 63	AD (moderate)	Rivastigmine + Memantine
#6	M, 78	AD (mild)	Donezepil
#7	M, 85	AD (moderate)	Rivastigmine + Memantine
#8	F, 81	AD (mild)	Rivastigmine
#9	F, 78	AD (mild)	Memantine
#10	M, 85	AD (mild)	Rivastigmine + Memantine
#11	F, 80	AD (mild)	Rivastigmine + Memantine
#13	F, 79	AD (mild)	Rivastigmine
#14	F, 82	AD (mild)	Donezepil
#16	F, 83	AD (moderate)	Donezepil + Memantine
#17	F, 63	AD (mild)	Donezepil
#18	F, 80	AD (mild)	Rivastigmine
#19	F, 80	AD (mild)	Rivastigmine
#20	M, 87	AD (mild)	Memantine
#21	M, 81	AD (mild)	Rivastigmine
#22	M, 87	AD (mild)	Donezepil
#23	F, 75	AD (mild)	Rivastigmine
#24	F, 75	AD (mild)	Rivastigmine
#25	F, 83	AD (mild)	Rivastigmine
#26	F, 84	AD (moderate)	Rivastigmine
#27	F, 81	AD (mild)	Rivastigmine
#28	F, 84	AD (moderate)	Memantine
#29	F, 92	AD (moderate)	Memenatine
#30	M, 86	AD (moderate)	Memenatine
#31	M, 77	AD (moderate)	Donezepil
#32	F, 88	AD (moderate)	Memantine
#33	F, 81	AD (moderate)	Rivastigmine
#34	F, 77	AD (moderate)	Rivastigmine + Memantine
#35	M, 87	AD (mild)	Memantine
#36	M, 84	AD (mild)	Memantine
#37	F, 77	AD (mild)	Rivastigmine
#38	F, 78	AD (mild)	Rivastigmine

Table S2. UniProt-KB code, experimental and theoretical average mass values ± standard deviations (SD), average (Mav) and monoisotopic ($[M+H]^+$), and elution times of proteins and peptides analyzed. Table reported also the m/z values and charge of the multiply-charged ions selected for XIC search in HPLC-low resolution MS, and those ones used for high-resolution MS/MS characterization.

Proteins/peptides	El. time (min ± 0.5)	Exper. (theor) Mav ± SD	m/z (charge) for XIC search	Exper. (theor) $[M+H]^+$ ± SD	m/z (charge) for MS/MS	PTMs
**Acid Proline-Rich Proteins**						
**PRP-1 2P (P02810)**	22.2	15515 ± 2 (15514-15515)	1293.9(+12), 1194.4(+13), 1035.3(+15), 970.7(+16), 913.6(+17)	15506.1± 0.1 (15506.24-15507.22)	1293.19(+12); 1034.75(+15); 970.14(+16); 862.46(+18)	N-Term(Gln->pyro-Glu), S_8(Phospho), S_{22}(Phospho)
**PRP-1 1P**	22.9	15435 ± 2 (15434-15435)	1287.2(+12), 1188.3(+13), 1030.0(+15), 965.7(+16), 908.9(+17)	15426.3 ± 0.1 (15426.27-15427.26)	1286.52(+12); 1187.64 (+13); 1029.42(+15)	N-Term(Gln->pyro-Glu), S_8 or S_{22}(Phospho)
**PRP-1 0P**	23.2	15355 ± 2 (15354-15355)	1280.5(+12), 1182.1(+13), 1024.6(+15), 960.7(+16), 904.2(+17)	15346.3± 0.1 (15346.31-15347.29)	903.72(+17); 853.57(+18)	N-Term(Gln->pyro-Glu)
**PRP-1 3P**	21.6	15595 ± 2 (15594-15595)	1418.7(+11), 1300.5(+12), 1200.6(+13), 1040.6(+15), 975.7(+16)	15586.3± 0.1 (15586.21-15587.19)	1732.80(+9);1114.30(+14); 1040.08(+25); 866.90 (+18)	N-Term(Gln->pyro-Glu) S_8, S_{17}, S_{22} (Phospho)
**PRP-3 2P (P02810)**	22.8	11161 ± 1 (11161-11162)	1595.5(+7), 1396.2(+8), 1015.7(+11), 931.1(+12), 859.6(+13)	11155.10± 0.07 (11156.08-11157.06)	1015.19(+11); 930.67(+12); 797.86(+14); 744.74(+15)	N-Term(Gln->pyro-Glu) S_8, S_{22} (Phospho) Fragment 1-106 of PRP-1
**PRP-3 1P**	23.4	11081 ± 1 (11081-11082)	1584.1(+7), 1386.2(+8), 1008.4(+11), 924.5(+12), 853.4(+13)	11076.00± 0.07 (11076.11-11077.09)	1385.51(+8); 1008.61(+11); 924.01(+12); 853.01(+13)	N-Term(Gln->pyro-Glu) S_8 or S_{22} (Phospho)
**PRP-3 0P**	23.8	11001 ± 1 (11001-11002)	1376.2(+8), 1101.2(+10), 917.8(+12) 786.8(+14)	10996.01± 0.07 (10996.14-10997.13)	1000.56 (+11); 786.37 (+14)	N-Term(Gln->pyro-Glu)
**PRP-3 2P desR$_{106}$**	22.8	11004 ± 1 (11005-11006)	1573.2(+7), 1223.8(+9), 1001.5(+11), 847.6(+13)	10999.85± 0.07 (10999.97-11000.96)	1001.00 (+11); 917.66 (+12)	N-Term(Gln->pyro-Glu) S_8, S_{22} (Phospho), R_{106} removal
**P-C peptide (P02810)**	15.0	4370.9 ± 0.4 (4370.8)	1457.9(+3), 1093.7(+4)	4369.19 ± 0.02 (4369.18)	1093.05(+4)	Fragment 107-150 of PRP-1
**Statherin**						
**Statherin (P02808)**	29.2	5380.0 ± 0.5 (5379.7)	1794.2(+3), 1345.9(+4), 1076.9(+5)	5377.46 ± 0.03 (5377.45)	1345.12(+4)	S_2(Phospho); S_3(Phospho)
**Statherin 1P**	28.9	5299.9 ± 0.5 (5299.7)	1767.6(+3), 1325.9(+4), 1060.9(+5)	5297.50 ± 0.03 (5297.48)	1325.13(+4)	S_3(Phospho)
**Fr. des-F$_{43}$**	27.8	5232.4 ± 0.5 (5232.5)	1745.1(+3), 1309.1(+4), 1047.5(+5)	5230.38 ± 0.03 (5230.38)	1308.35(+4)	C-Term. F_{43}removal
**Fr. desT$_{42}$-F$_{43}$**	27.9	5131.2 ± 0.5 (5131.4)	1711.4(+3), 1283.8(+4), 1027.2(+5)	5129.33 ± 0.03 (5129.33)	1283.09(+4)	C-Term. T_{42}-F_{43}removal
**Fr. desD$_1$**	28.7	5264.7 ± 0.5 (5264.6)	1755.9(+3), 1317.2(+4), 1053.9(+5)	5262.41 ± 0.03 (5262.42)	1316.36(+4)	N-Term. D_1removal

Proteins/peptides	El. time (min ± 0.5)	Exper. (theor) Mav ± SD	m/z (charge) for XIC search	Exper. (theor) $[M+H]^+$ ± SD	m/z (charge) for MS/MS	PTMs
**Fr. des1-9**	28.5	4127.9 ± 0.4 (4127.6)	1376.9(+3), 1032.9(+4)	4125.99 ± 0.02 (4125.99)	1376.00(+3)	N-Term. 1-9 residue removal
**Fr. des1-10**	28.0	3971.3 ± 0.4 (3971.4)	1986.7(+2), 1324.8(+3)	3969.90 ± 0.01 (3969.89)	1323.97(+3)	N-Term. 1-10 residue removal
**Fr. des1-13**	27.5	3645.2 ± 0.4 (3645.0)	1823.6(+2), 1216.1(+3)	3643.68 ± 0.01 (3643.68)	1215.56(+3); 911.92(+4)	N-Term. 1-12 residue removal
			**P-B peptide**			
**P-B peptide (P02814)**	30.0	5792.9 ± 0.5 (5792.7)	1932.0(+3), 1449.2(+4), 1159.6(+5)	5790.06 ± 0.03 (5790.04)	965.85(+6)	N-Term(Gln->pyro-Glu)
**Fr. des1-4**	30.0	5371.0 ± 0.5 (5371.3)	1791.4(+3), 1343.8(+4), 1075.3(+5)	5368.84 ± 0.03 (5368.82)	1343.21(+4); 1074.77(+5)	N-Term. 1-4 residue removal
**Fr. des1-5**	30.3	5215.0 ± 0.5 (5215.1)	1739.4(+3), 1304.8(+4), 1044.0(+5)	5212.75 ± 0.03 (5212.73)	1303.94(+4)	N-Term. 1-5 residue removal
**Fr. des1-7**	30.1	5060.1 ± 0.5 (5060.9)	1688.0(+3), 1266.2(+4), 1013.2(+5)	5058.67 ± 0.03 (5058.65)	1265.42(+4)	N-Term. 1-7 residue removal
**Fr. des1-12**	27.5	4549.0 ± 0.5 (4549.3)	1517.5(+3), 1138.3(+4)	4547.10 ± 0.02 (4547.41)	1137.85(+4); 910.48(+5)	N-Term. 1-12 residue removal
**Histatins**						
**Hst-1 (P15515)**	21.9	4928.2 ± 0.5 (4928.2)	1644.1(+3), 1233.5(+4)	4926.21 ± 0.02 (4926.20)	704.61(+7)	S2(Phospho)
**Hst-1 0P**	22.0	4848.2 ± 0.5 (4848.2)	1617.4(+3), 1213.5(+4)	4846.24 ± 0.02 (4846.23)	693.18(+7)	
**Hst-3 (P15516)**	17.7	4062.2 ± 0.4 (4062.4)	1355.1(+3), 1016.6(+4)	4060.98 ± 0.02 (4060.98)	813.00(+5); 677.67(+6)	
**Hst-3 1/25**	14.3	3192.4 ± 0.3 (3192.5)	1065.1(+3), 799.1(+4)	3191.62 ± 0.01 (3191.62)	532.78(+6); 456.81(+7)	Fragment 1-25 of Hst-3
**Hst-3 1/24**	14.6	3036.5 ± 0.3 (3036.3)	1013.2(+3), 760.1(+4)	3035.53 ± 0.01 (3035.52)	607.91(+5); 506.76(+6)	Fragment 1-24 of Hst-3
			**Cystatins**			
**A (P01040)**	31.8	11005.354 ± 2 (11006.5)	1001.59(+11), 1101.59(+10), 1223.94(+9), 1376.81(+8), 1573.36(+7), 1835.42(+6)	11000.65± 0.07 (11000.67)	1375.96 (+8); 847.13 (+13); 786.69 (+14)	
**A Nα-Ac.**	33	11047.43 ± 2 (11048.5)	1005.41(+11), 1105.85(+10), 1228.61(+9), 1382.06(+8), 1579.36(+7), 1842.42(+6)	11042.55± 0.07 (11042.68)	1381.21 (+8); 1227.85 (+9); 789.69 (+14)	N-Term.-α-Acetylation
**B-SSG(P04080)**	32.8	11485.8 ± 2 (11486.9)	1915.5(+6), 1642.0(+7), 1436.9(+8), 1277.3(+9), 1149.7(+10), 1045.3(+11)	11480.69± 0.07 (11480.68)	1149.58 (+10); 1045.25 (+11)	C_3Glutathionylation

Proteins/peptides	El. time (min ± 0.5)	Exper. (theor) Mav ± SD	m/z (charge) for XIC search	Exper. (theor) $[M+H]^+$ ± SD	m/z (charge) for MS/MS	PTMs
**B-SSC**	32.9	11299.8 ± 2 (11300.7)	1884.5(+6), 1615.4(+7), 1413.6(+8), 1256.7(+9), 1131.1(+10), 1028.6(+11)	11294.54 ± 0.07 (11294.61)	-	C_3Cysteinylation
**B S-S dimer**	34.3	22358 ± 2 (22361.3)	1862.4(+12), 1721.1(+13), 1598.2(+14), 1491.8(+15), 1398.6(+16), 1316.4(+17), 1243.3(+18), 1177.9(+19), 1119.1(+20), 1065.8(+21), 1017.4(+22), 973.2(+23)	nd		1 interchaindisulfide bridge
**C (P01034)**	35.1	13342 ± 2 (13343.1)	1483.57(+9), 1335.32(+10), 1214.02(+11), 1112.93(+12), 1027.40(+13)	13335.32 ± 0.08 (13335.58)	1112.21 (+12); 834.41 (+16)	2 intrachaindisulfidebridges
**D-R_{26} des1-5 (P28325)**	37.7	13517 ± 2 (13517.3)	1690.70(+8), 1502.90(+9), 1352.70 (+10), 1229.80 (+11), 1127.4 (+12), 1040.40 (+13)	13509.43± 0.08 (13509.65)	1501.96 (+9); 1351.86 (+10); 965.90 (+14)	N-Term(Gln->pyro-Glu) after 1-5 residue removal, 2 intrachain disulfi bridges
			**Cystatins S-type**			
**S1 (P01036)**	35.3	14266 ± 2 (14265)	1784.3(+8), 1586.1(+9), 1427.6(+10), 1297.9(+11), 1189.8(+12), 1098.4(+13), 1020.0(+14)	14256.66± 0.09 (14256.77)	1296.98 (+11);1097.60 (+13);1019.27(+14)	S_3(Phospo) on cystatin S, 2 intrachain disulfide bridges
**S1ox**	35.3	14281 ± 2 (14280.7)	1786.40(+8), 1589.70 (+9), 1429.30 (+10), 1299.50 (+11), 1191.30 (+12), 1099.70 (+13)	14272.66 ± 0.09(14272.77)	1428.18 (+10); 1298.43 (+11);1190.31 (+12);892.99 (+16)	S_3(Phospo), W_{23} oxidation, 2 intrachaindisulfide bridges
**S2**	35.3	14346 ± 2 (14345)	1794.3(+8), 1595.0(+9), 1435.6(+10), 1305.2(+11), 1196.5(+12), 1104.5(+13), 1025.7(+14)	14336.58 ± 0.09 (14336.74)	1434.57 (+10); 1195.65 (+12); 896.98 (+16)	S_1, S_3 (di-Phospho) on cystatin S, 2 intrachain disulfide bridges
**SN (P01037)**	34.6	14312 ± 2 (14313)	1790.0(+8), 1591.2(+9), 1432.2(+10), 1302.1(+11), 1193.7(+12), 1101.9(+13), 1023.3(+14)	13304.03 ± 0.09 (13304.09)	951.22 (+14); 740.06 (+18)	2 intrachaindisulfide bridges
**SNox**	34.6	14328 ± 2 (14328)	1792.30(+8), 1593.20 (+9), 1434.00 (+10), 1303.30 (+11), 1195.20 (+12), 1103.30 (+13)	14320.10± 0.09 (14320.09)	1302.74 (+11);1194.26 (+12);1102.47 (+13); 955.61 (+15);	W_{23}oxidation
**SA (P09228)**	36.8	14347 ± 2 (14346)	1794.4(+8), 1595.1(+9), 1435.7(+10), 1305.3(+11), 1196.6(+12), 1104.6(+13), 1025.8(+14)	14338.02± 0.09 (14338.01)	1103.85 (+13); 1025.07 (+14); 956.80 (+15); 897.06 (+16); 844.35 (+17); 797.50 (+18)	1 intrachaindisulfide bridge
			**Antileukoproteinase**			
**SLPI (P03973)**	26.2	11702.2 ± 1 (11706)	1952.64(+6), 1673.84(+7), 1464.73(+8), 1302.10(+9)	11703.29 ± 0.07 (11702.36)	1301.26(+9); 1171.24(+10)	8 intrachain disulfide bridges
			**α-Defensins**			
**α-defensin 1 (P59665)**	23.5	3442.5 ± 2 (3442.1)	1772.03(+2), 1148.36(+3), 861.52(+4)	3440.45 ± 0.01 (3440.52)	861.13(+4); 689.10(+5)	2 intrachain disulfide bridges
**α-defensin 2 (P59665/6)**	23.5	3370.4 ± 1 (3370.9)	1686.49(+2), 1124.66(+3), 843.75(+4)	3370.41 ± 0.01 (3370.44)	1124.48(+3); 843.61(+4); 675.09(+5)	2 intrachain disulfide bridges
**α-defensin 3 (P59666)**	23.5	3485 ± 2 (3486.1)	1744.03(+2), 1163.03(+3), 872.52(+4)	3484.53 ± 0.01 (3484.51)	872.13(+4)	2 intrachain disulfide bridges

Proteins/peptides	El. time (min ± 0.5)	Exper. (theor) Mav ± SD	m/z (charge) for XIC search	Exper. (theor) $[M+H]^+$ ± SD	m/z (charge) for MS/MS	PTMs
**α-defensin 4 (P12838)**	27.2	33708 ± 1 (3709.4)	1855.71(+2), 1237.48(+3), 928.36(+4)	3707.68 ± 0.01 (3707.77)	927.94(+4)	2 intrachain disulfide bridges
			**Thymosins β4**			
**Tβ4 (P62328)**	18.5	4964.0± 1 (4963.5)	1655.51(+3), 1241.88(+4), 993.71(+5)	4961.50 ± 0.02 (4961.48)	877.76(+6)	N-Term.-α-Acetylation
			**S100A proteins**			
**S100A12 (P80511)**	40.0	10444 ± 2 (10443.9)	1306.5(+8), 1161.4(+9), 1045.4(+10), 950.4(+11)	10438.74±0.07 (10438.49)	1044.75 (+10);803.88 (+13); 696.83 (+15)	M_1 removal
**S100A7 (P31151)**	37.0	11367 ± 2 (11367.8)	1422.0(+8), 1264.1(+9), 1137.8(+10), 1034.4(+11)	11360.43 ± 0.07(11361.52)	1263.28 (+9); 1033.77 (+11); 947.71 (+12); 874.89 (+13)	M_1 removal, N-Term.-α-Acetylation, D_{27} variant
**S100A8(P05109)**	40.4	10833 ± 2 (10834.5)	1355.3(+8), 1204.8(+9), 1084.5(+10), 985.9(+11)	10826.60 ± 0.07 (10828.657)	985.42(+11); 903.39(+12); 833.97(+13)	
**S100A8 hyperoxidized**	39.3	10915 ± 2 (10914.6)	1365.3(+8), 1213.7(+9), 1092.5(+10), 993.2(+11)	10908.40 ± 0.07 (10908.631)	840.05 (+13)	C_{42}-SO_3H and W_{54}dioxidation or C_{42}-SO_3H and W_{54} oxidation and $M_{1/78}$oxidation
**S100A8 SNO**	40.8	10863 ± 2 (10863.5)	1358.9(+8), 1208.1(+9), 1087.3(+10), 988.6(+11)	10858.67 ± 0.07(10857.647)	836.13 (+13);776.47 (+14); 724.78 (+15)	C_{42}nitrosylation
**S100A9(S) (P06702)**	42.2	12690 ± 2 (12689.2)	1410.9(+9), 1269.9(+10), 1154.6(+11), 1058.4(+12), 977.1(+13)	12682.21 ± 0.08 (12682.293)	1410.03 (+9); 1269.13 (+10); 1057.77 (+12); 976.48 (+13); 846.42 (+15); 705.52 (+18)	N-Term.-α-Acetylation after 1-5 residue removal
**S100A9(S) 1P**	42.2	12770 ± 2 (12769.2)	1419.8(+9), 1277.9(+10), 1161.8(+11), 1065.1(+12), 983.3(+13)	12762.05 ± 0.08 (12762.26)	912.96 (+14); 852.29 (+15); 798.96 (+16)	N-Term.-α-Acetylation after 1-5 residue removal, T_{108} (Phospho)
**S100A9(S) M-ox**	41.3	12706 ± 2 (12705.2)	1412.7(+9), 1271.5(+10), 1156.0(+11), 1059.8(+12), 978.3(+13)	12698.39 ± 0.08 (12698.288)	907.95 (+14); 847.49 (+15)	N-Term.-α-Acetylation after 1-5 residue removal,$M_{89 \text{ or } 78 \text{ or } 76 \text{ or } 58}$ oxidation
**S100A9(S) M-ox 1P**	41.3	12786 ± 2 (12785.2)	1421.9(+9), 1279.5(+10), 1163.3(+11), 1066.4(+12), 984.5(+13)	12778.21 ± 0.08 (12778.255)	1420.70 (+9); 983.87 (+13); 913.66 (+14)	N-Term.-α-Acetylation after 1-5 residue removal, T_{108} (Phospho), $M_{89 \text{ or } 78 \text{ or } 76 \text{ or } 58}$ oxidation
**S100A9(L) SSG**	41.5	13459 ± 2 (13458.1)	1346.8(+10), 1224.5(+11), 1122.5(+12), 1036.3(+13), 962.3(+14)	13450.51 ± 0.08 (13450.55)	1035.58 (+13); 961.68 (+14);841.60 (+16);792.15 (+17); 748.20 (+18)	M_1 removal, N-Term.-α-Acetylation, C_2glutathionylation

Table S3: XIC peak areas values (mean ± SD) normalized on TPC and frequencies of proteins/peptides resulted with similar levels between AD group and HC group. (•), not statistically significant p-values. (NA), absence of a protein.

Protein/peptide	XIC Peak Areas x 10^5(mean ± SD) and Frequency		p-values
	**AD**	**HC**	**AD vs HC**
PRP-1 2P	37.6 ± 27.2 (34/35)	28.8 ± 18.7 (34/34)	•
PRP-1 1P	5.0 ± 4.3 (34/35)	3.8 ± 3.5 (31/34)	•
PRP-1 0P	0.2 ± 0.6 (8/35)	0.3 ± 0.3 (19/34)	•
PRP-1 3P	0.09 ± 0.2 (8/35)	0.3 ± 0.4 (16/34)	•
PRP-3 2P	13.0 ± 10.4 (35/35)	9.4 ± 6.7 (34/34)	•
PRP-3 1P	2.1 ± 1.9 (35/35)	1.5 ± 1.1 (32/34)	•
PRP-3 0P	0.06 ± 0.1 (10/35)	0.04 ± 0.07 (9/34)	•
PRP-3 2P, desR$_{106}$	1.8 ± 1.8 (31/35)	2.4 ± 2.2 (28/34)	•
Stath. 1P	0.3 ± 0.4 (29/35)	0.3 ± 0.2 (24/34)	•
Stath. desT$_{42}$-F$_{43}$	0.4± 0.4 (26/35)	0.2 ± 0.2 (30/34)	•
Stath. desD$_1$	0.4 ± 0.5 (32/35)	0.3 ± 0.3 (30/34)	•
Stath. des1-10	0.3 ± 0.3 (26/35)	0.2 ± 0.2 (27/34)	•
P-B peptide	7.8 ± 6.6 (35/35)	6.4 ± 4.4 (34/34)	•
P-B des1-4	0.6± 0.8 (2/35)	0.7 ± 0.6 (32/34)	•
P-B des1-5	0.8 ± 0.8 (34/35)	1.0 ± 1.1 (34/34)	•
P-B des1-7	1.3 ± 2.3 (34/35)	0.8 ± 0.5 (34/34)	•
P-B des1-12	0.7 ± 0.7 (34/35)	0.8 ± 1.3 (31/34)	•
SLPI	0.1 ± 0.2 (13/35)	0.06 ± 0.09 (13/34)	•
Hst-3	0.6 ± 1.0 (18/35)	0.4 ± 0.7 (18/34)	•
Hst-3 1/25	0.2 ± 0.3(9/35)	0.2 ± 0.3 (17/34)	•
Hst-3 1/24	1.1 ± 1.5 (20/35)	0.6 ± 0.9 (10/34)	•
Cyst A Nα-Ac	0.2 ± 0.2 (24/35)	0.2 ± 0.5 (23/34)	•
Cyst. C	0.1 ± 0.4 (6/35)	0.08 ± 0.2 (5/34)	•
Cyst. D-R$_{26}$ des1-5	0.3 ± 0.4 (13/35)	0.3 ± 0.4 (16/34)	•
Cyst S1 tot	6.4 ± 8.8 (31/35)	4.1 ± 3.7 (30/34)	•
Cyst. S2	1.6 ± 2.6 (20/35)	1.2 ± 1.2 (26/34)	•
Cyst. SN tot	12.3 ± 21.3 (32/35)	6.7 ± 6.6 (29/34)	•
Cyst. SA	1.4 ± 3.4 (8/35)	0.5 ± 1.2 (7/34)	•
S100A7	0.3 ± 0.7 (13/35)	0.2 ± 0.3 (11/34)	•

Table S4: XIC peak areas values (mean ± SD) normalized on TPC, frequencies and p-values obtained by statistical analysis by comparing the three patients' groups treated with different therapies by non-parametric ANOVA with the Krustal-Wallis test and Dunn's post test. p-values > 0.05 are not statistically significant (•).

Peptide	XIC Peak Areas x 10^5(mean ± SD) and Frequency				ANOVA p value
	G1 (nr 19)	G2 (nr 8)	G3 (nr 8)	HC (nr 34)	
α-defensin 1	2.2 ± 2.3 (19)	2.3 ± 1.8 (8)	0.6 ± 0.8 (5)	0.5 ± 0.6 (24)	p < 0.0001 (G1vs G3*, G2 vs G3*, G1 vs HC***, G2 vs HC**)
α-defensin 2	1.5 ± 1.5 (19)	1.5 ± 1.2 (7)	0.4 ± 0.6 (5)	0.4 ± 0.4 (27)	p = 0.0003 (G1vs G3*, G1 vs HC**, G2 vs HC*)
α-defensin 3	0.7 ± 0.5 (16)	0.8 ± 0.5 (8)	0.2 ± 0.4 (2)	0.2 ± 0.3 (17)	p < 0.0001 (G1vs G3*, G2 vs G3*, G1 vs HC***, G2 vs HC**)
α-defensin 4	0.2 ± 0.3 (8)	0.4 ± 0.2 (8)	0.04 ± 0.08 (2)	0.05 ± 0.1 (11)	p = 0.0004 (G1vs G2*, G2 vs G3**, G2 vs HC***)
Tβ4	0.5 ± 0.6 (14)	1.1 ± 0.9 (8)	0.2 ± 0.4 (3)	0.2 ± 0.4 (16)	p = 0.002 (G2vs G3**, G2 vs HC**)
Stath. 2P	8.8 ± 7.7 (19)	5.9 ± 4.4 (6)	11.0 ± 7.1 (8)	1.1 ± 0.9 (33)	p < 0.0001 (G1 vs HC***, G2vs HC*, G3 vs HC***)
Stath. Des1-9	0.5 ± 0.6 (16)	0.6 ± 0.9 (6)	0.8 ± 0.6 (7)	0.08 ± 0.1 (24)	p = 0.0002 (G1 vs HC**, G3 vs HC**)
P-C peptide	9.3 ± 5.1 (19)	8.6 ± 12.6 (6)	12.3 ± 6.9 (8)	5.6 ± 4.4 (34)	p = 0.01 (G3 vs HC*)
Cyst. B tot	2.0 ± 2.2 (18)	1.9 ± 1.6 (8)	0.9 ± 1.2 (7)	9.3 ± 5.1 (27)	p = 0.006 (G1vs HC*, G2 vs HC*)
Cyst A	1.8 ± 1.6 (17)	2.9 ± 1.9 (8)	1.2 ± 0.9 (7)	1.0 ± 0.9 (29)	p = 0.02 (G2 vs HC*)
S100A8 tot	1.1 ± 1.5 (12)	0.9 ± 1.1 (6)	0.4 ± 0.5 (4)	0.08 ± 0.2(5)	p < 0.0001 (G1 vs HC***, G2 vs HC**)
S100A8-SNO	1.1 ± 2.3 (9)	0.7 ± 1.0 (3)	Not detected	Not detected	p < 0.0001 (G1 vs G3*, G1 vs HC***)
S100A9(S) tot	1.1 ± 1.5 (18)	0.9 ± 1.1 (7)	0.4 ± 0.5 (7)	0. .5 ± 0.8 (16)	p < 0.0001 (G1 vs HC***, G2 vs HC*)

2 Supplementary Figures

Supplementary Figure S1. Immune-detection analysis of the total α-defensins by dot-blotting of a triplicate of a salivary pools of AD samples and HC samples (panel A), statistical analysis of the normalized signal intensity of the AD and HC spots (panel B). Normalization performed on cystatin SN signal (panel C). Statistical comparison between SN signals in AD and HC(panel D).

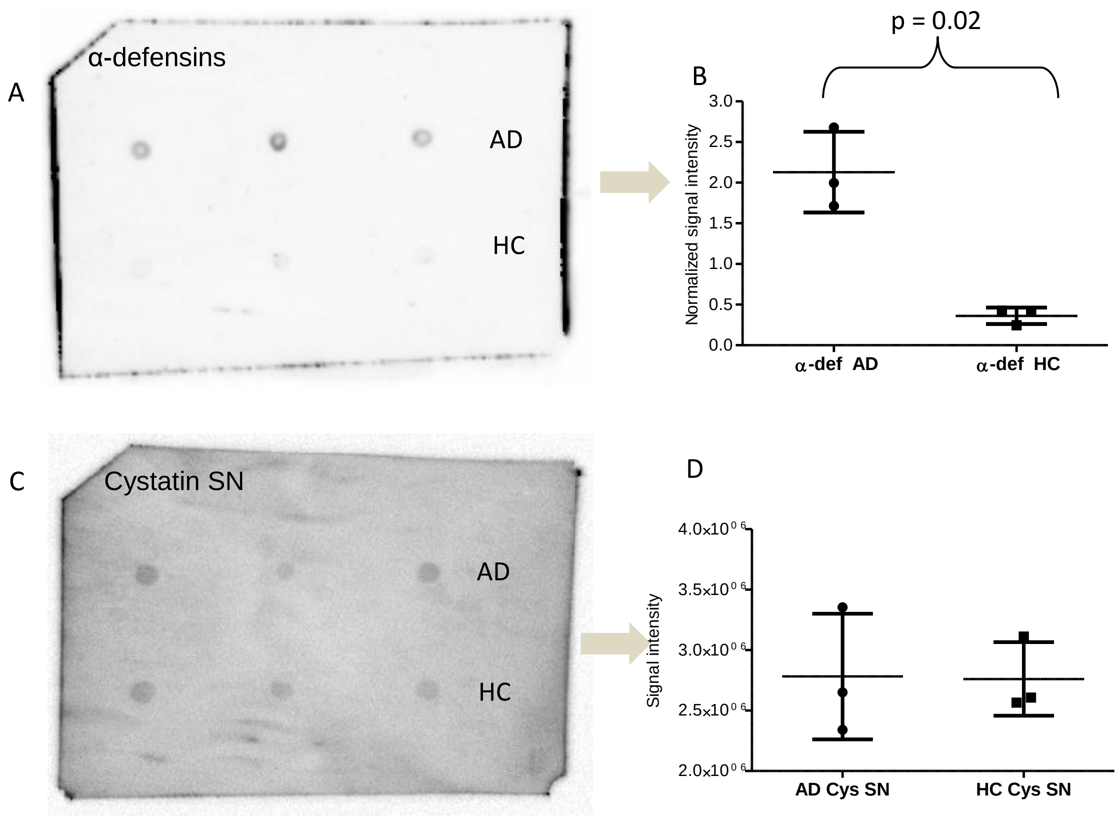

Supplementary Figure S2. Immune-detection analysis of the total S100A8 by dot-blotting of a triplicate of a salivary pools of AD samples and HC samples (panel A), statistical analysis of the normalized signal intensity of the AD and HC spots (panel B). Normalization performed on cystatin SN signal (panel C). Statistical comparison between SN signals in AD and HC(panel D).

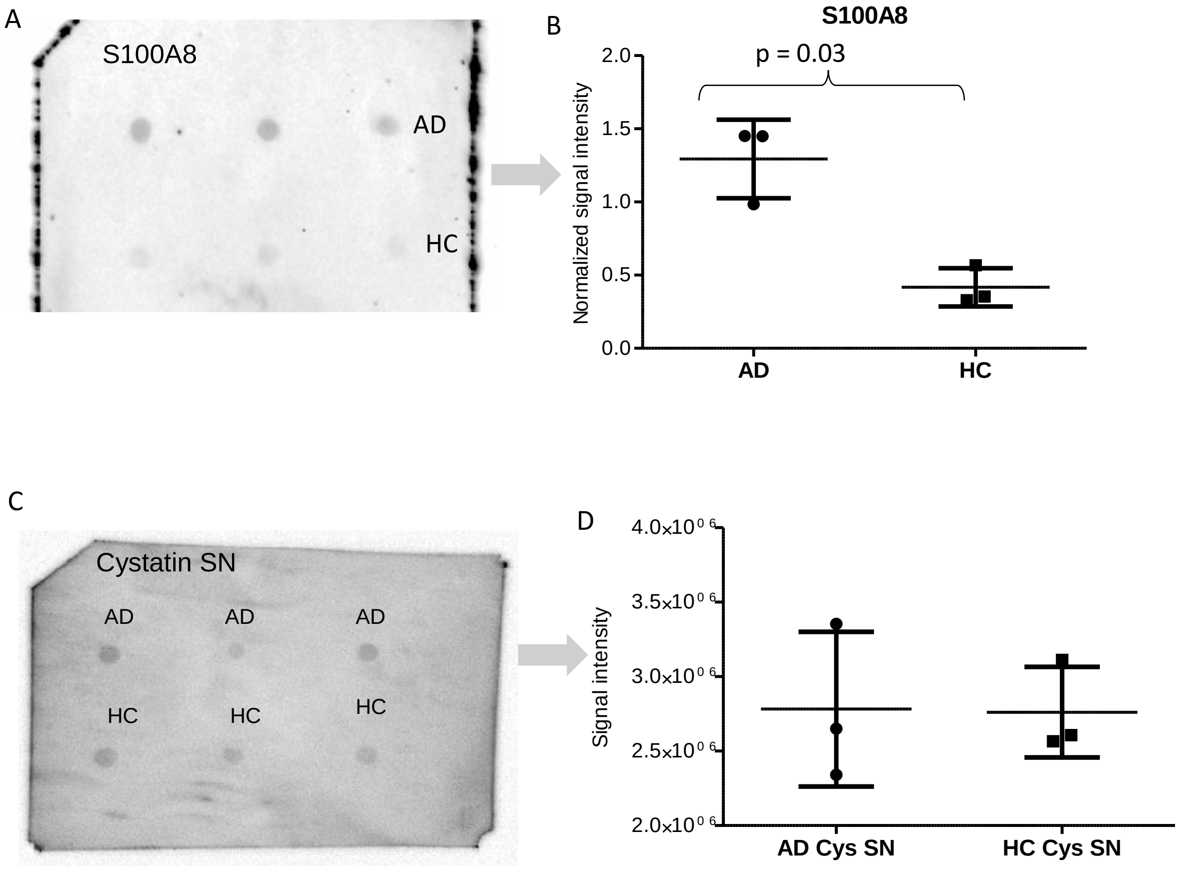

Supplementary Figure S3. Immune-detection analysis of the total S100A9 by dot-blotting of a triplicate of a salivary pools of AD samples and HC samples (panel A), statistical analysis of the normalized signal intensity of the AD and HC spots (panel B). Normalization performed on cystatin SN signal (panel C). Statistical comparison between SN signals in AD and HC(panel D).

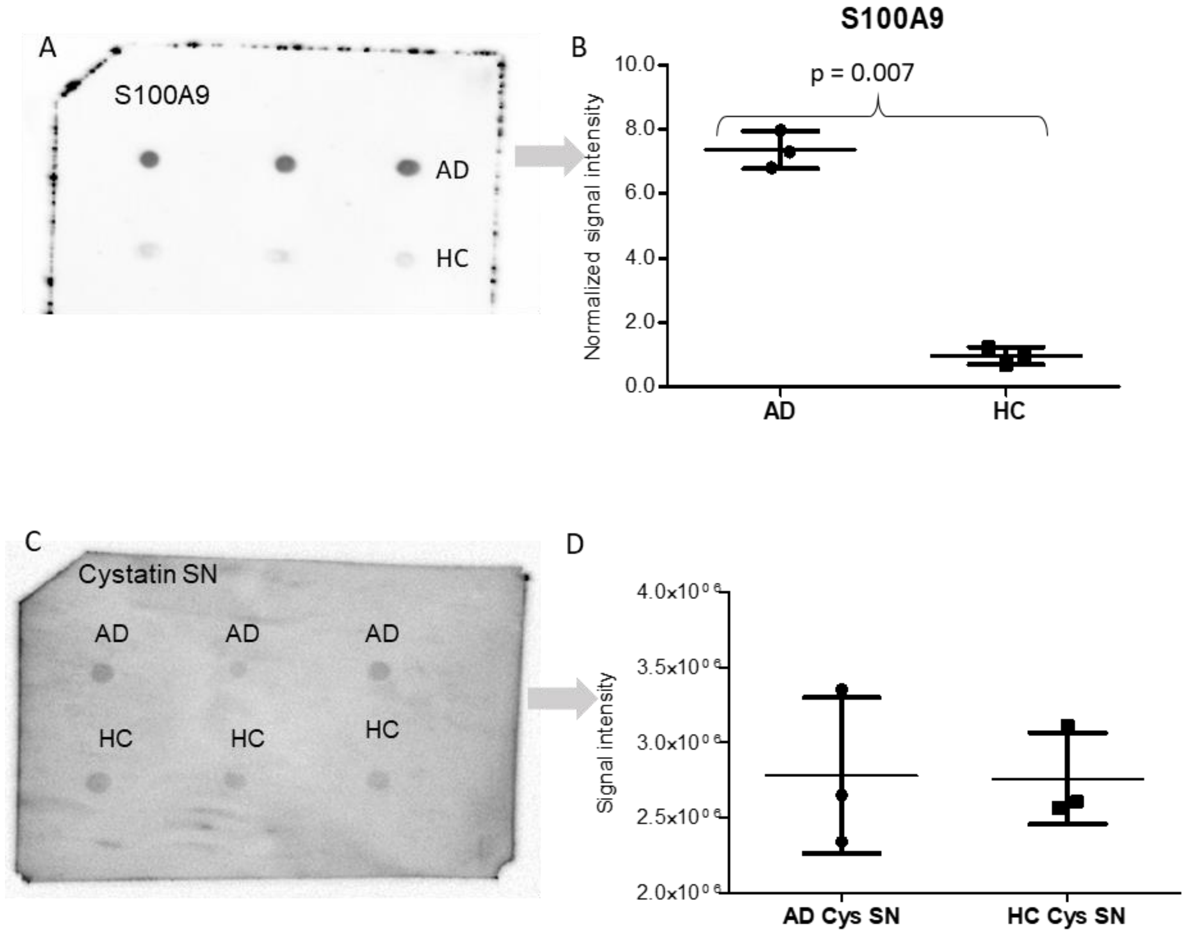

Supplementary Figure S4. Immune-detection analysis of Tβ4 by dot-blotting of a triplicate of a salivary pools of AD samples and HC samples (panel A), statistical analysis of the normalized signal intensity of the AD and HC spots (panel B). Normalization performed on the signal of the standard Tβ4 0.26 nmol (panel C).

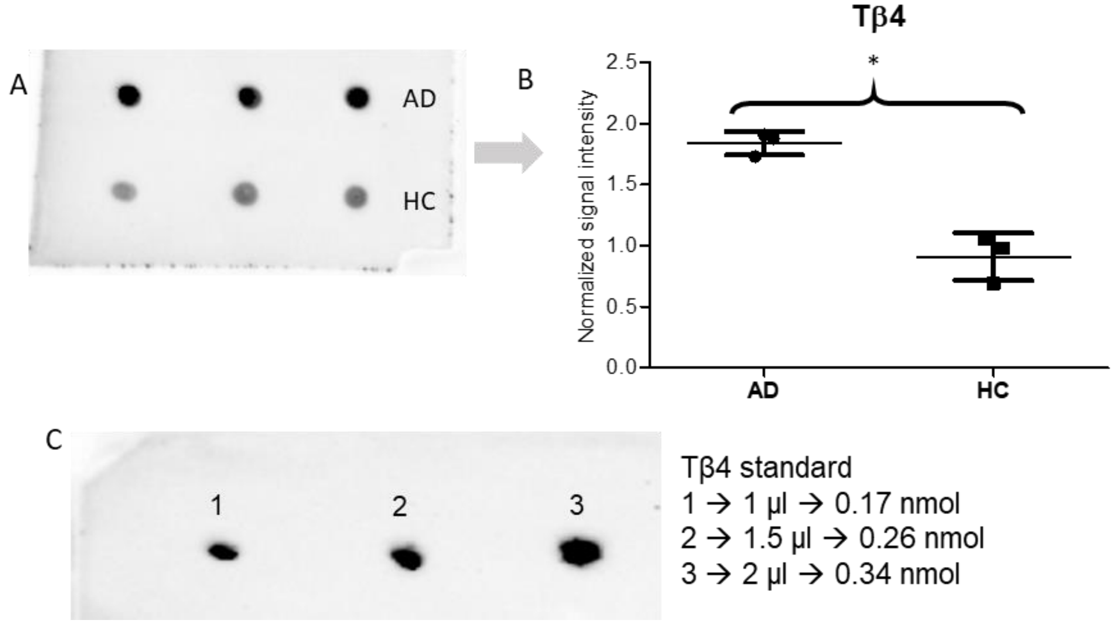

Supplementary Figure S5. Immune-detection analysis of cystatin B by dot-blotting of a triplicate of a salivary pools of HC samples and AD samples, and of the HPLC-enriched fraction of cystatin B prepared form human saliva, blotted at different amounts indicated as 1, 2, and 3, (panel A). Statistical analysis of the normalized signal intensity of the AD and HC spots with respect the standard 2 at 0.2 µg of total proteins (panel B).

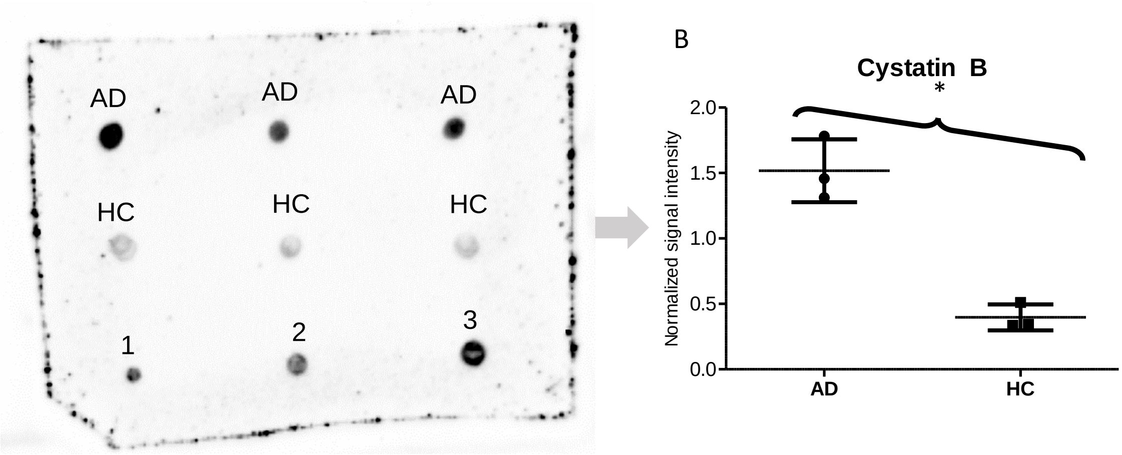

Enriched cystatin B fraction from human saliva

1 → 0.5 µl → 0.1 µg of total proteins

2 → 1 µl → 0.2 µg of total proteins

3 → 2 µl → 0.4 µg of total proteins

Supplementary Fig. S6. Total protein concentration measured in the subgroups of patients with different pharmacological treatment.

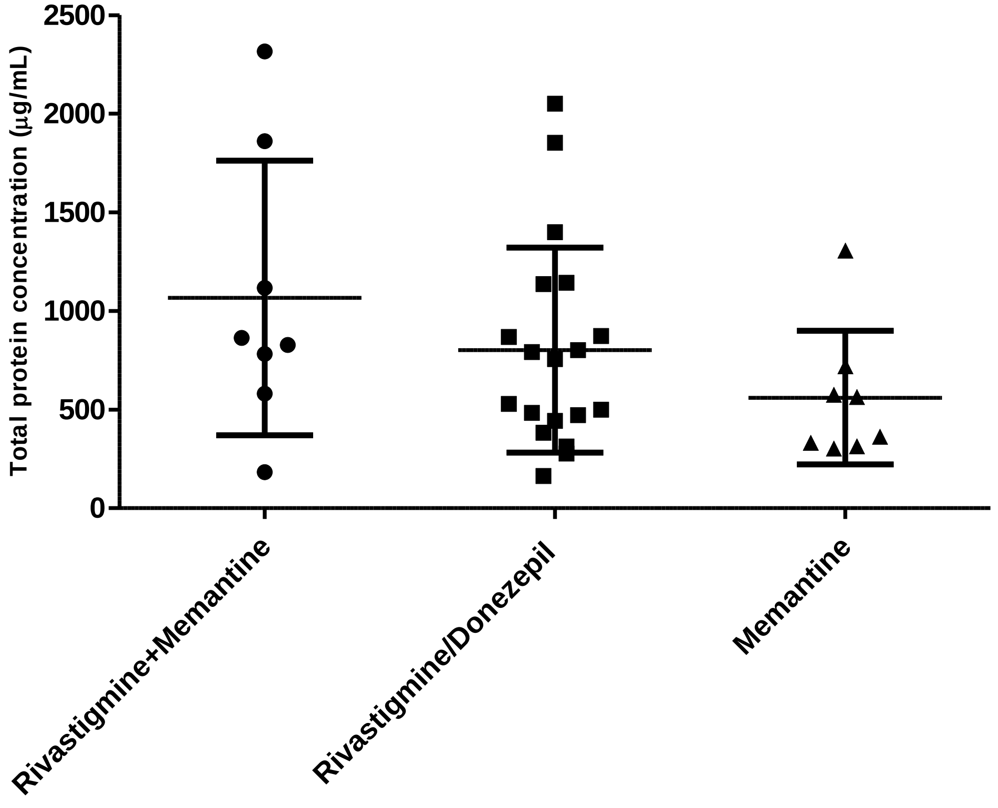

Supplement: Supplementary file 1 [file Data_Sheet_1.pdf]
